# Long-term systolic blood pressure and cardiovascular risks among patients with ischemic stroke: a register-based cohort study

**DOI:** 10.7189/jogh.15.04321

**Published:** 2025-11-07

**Authors:** Chunbao Mo, Xia Li, Shuang Wang, Jiangshui Wang, Li He, Ruiyang Peng, Jing Zheng, Fengchao Liang, Dongfeng Gu

**Affiliations:** 1 School of Public Health and Emergency Management, School of Medicine, Southern University of Science and Technology, Shenzhen, China; 2 Shenzhen Key Laboratory of Cardiovascular Health and Precision Medicine, Southern University of Science and Technology, Shenzhen, China; 3 Shenzhen Health Development Research and Data Management Center, Shenzhen, China; 4 Department of Epidemiology, Fuwai Hospital, National Center for Cardiovascular Diseases, Chinese Academy of Medical Sciences and Peking Union Medical College, Beijing, China

## Abstract

**Background:**

The impact of blood pressure fluctuations on the prognosis of stroke has been well documented, but little is known about the association between long-term systolic blood pressure (SBP) levels and the risks of cardiovascular outcomes in patients with ischemic stroke (IS).

**Methods:**

In this retrospective cohort study, we included a total of 11 357 eligible IS patients hospitalised in Shenzhen, China between 1 July 2017 and 1 October 2023. One-year levels of SBP after IS patient discharge were identified using group-based trajectory models (GBTM). Propensity score-overlap weighted Cox regression models were used to assess the associations between SBP levels and the risks of recurrent stroke and major adverse cardiovascular events (MACE; including recurrent stroke, ischemic heart disease, and heart failure) within a 36-month follow-up period, respectively. Furthermore, we quantitatively assessed the benefits potentially gained from optimal SBP levels by calculating age-scale restricted mean survival times.

**Results:**

Three one-year GBTM-derived SBP level patterns were identified: normal (n = 2120), high-normal (n = 7949), and uncontrolled SBP (n = 1288). During a median follow-up of 1.75 years, IS patients with normal and high-normal SBP were associated with lower risks of recurrent stroke or MACE, with weighted hazard ratios (95% confidence interval (CI)) ranging from 0.68 (95% CI = 0.54–0.86) to 0.89 (95% CI = 0.78–1.02), compared to those with uncontrolled SBP. Furthermore, IS patients aged 45 to 70 years with normal or high-normal SBP may derive greater health benefits, with the event-free survival time ranging from 7.12 to 0.27 years.

**Conclusions:**

Maintaining sustained normal or high-normal SBP levels one year after discharge may be associated with a reduced risk of adverse cardiovascular events and potentially yields greater health benefits for IS patients.

Stroke is a significant contributor to disability and mortality and ranks as the third leading cause of death globally [1]. According to estimates from the Global Burden of Disease study [2], ischemic stroke (IS) alone accounted for 7.3 million deaths worldwide in 2021, representing 50.8% of total stroke-related deaths, of which 35.7% occurred in China. Statistics from a national survey reveal that there are approximately 2.5 million new cases of stroke in China each year, of which 60% to 80% are IS [3]. Moreover, stroke remained the third leading cause of death in China in 2019 [4]. These statistics highlight that a national prioritisation of stroke prevention and management worldwide is urgently needed [5].

Elevated blood pressure (BP) or hypertension is a risk factor that significantly increases the risk of adverse outcomes of IS. Consequently, prioritising the effective management of BP becomes crucial for mitigating recurrence risks, enhancing prognosis, and elevating the quality of life for IS patients [6]. Systolic BP (SBP) is an independent risk predictor for stroke and demonstrates a stronger predictive performance compared to diastolic BP (DBP) [7]. In addition to the baseline SBP levels, the dynamic levels of SBP are also closely associated with the prognosis in patients with IS. For example, maintaining a high-SBP trajectory (mean value (x̄) = 160 mm Hg) within 24 hours of IS onset may presage an unfavourable outcome, whereas maintaining a stable level of SBP at a lower threshold (x̄ = 137 mm Hg) may be associated with more favourable prognoses among IS survivors [8,9]. Although the relationship between SBP fluctuations and the prognosis of IS has been well established [8,9], evidence regarding the impact of long-term SBP levels after discharge on cardiovascular outcomes and relevant health benefits gained from optimal SBP levels in patients with IS are relatively limited. Moreover, the management and rehabilitation of IS patients are long-term processes, assessing the health benefits derived from different long-term SBP levels across different age groups may provide more clinically relevant insights [10].

To assess the impact of long-term SBP levels on the prognosis of IS patients, we conducted a register-based retrospective cohort study in Shenzhen, China to investigate the association between one-year SBP levels after hospital discharge and the risks of adverse cardiovascular outcomes, as well as to estimate the health benefits gained from optimal SBP levels in IS patients.

## METHODS

### Sources of data

We conducted this retrospective cohort study using the Population Health Informatization Platform [11–13], a database in Shenzhen, China, that integrates health-related data, including demographics, medical records (outpatient and inpatient), therapies records, chronic disease management records, and death registration records. BP data were primarily retrieved from chronic disease management records, and secondarily from data on outpatient visits or hospitalisations. All datasets were linked using encrypted unique IDs [11–13].

### Study population

Patients hospitalised with IS (International Classification of Diseases 10th Revision, ICD-10 code: I63) as the primary cause between 1 July 2017 and 1 October 2023 were investigated. If a patient had multiple admissions during this period, only the first admission with a stroke diagnosis was considered as the earliest time of onset. Initially, we included 15 772 patients who had at least three BP measurements within one year after IS discharge, and further excluded those who:

1) were not registered as permanent residents (n = 967);

2) were diagnosed with both ischemic and haemorrhagic stroke simultaneously (n = 202);

3) were aged <45 or >95 years (n = 1048), as stroke predominantly affects middle-aged and elderly populations, and extreme older age is frequently accompanied by multiple comorbidities [11];

4) experienced recurrent stroke, major adverse cardiovascular events (MACE), or died within 12 months after the first diagnosis of IS (n = 1926) [14]; or

5) had an observation period of less than 12 months after discharge (n = 272). Finally, a total of 11 357 eligible participants were included in the present study (Figure S1 in the **Online Supplementary Document**). This study was approved by the ethical review committee of Southern University of Science and Technology (NO.20210067).

### BP measurement and long-term BP levels

**Figure 1:**
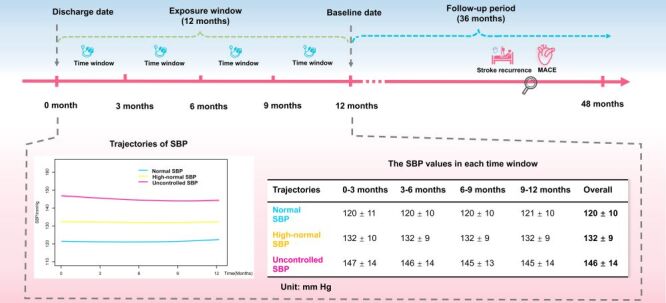
Study design diagram for the association between long-term SBP levels and clinical outcomes. Long-term SBP groups were constructed based on the latent clusters identified using the group-based trajectory model. MACE – major adverse cardiovascular events, SBP – systolic blood pressure.

To capture long-term SBP levels for each IS patient, we defined the 12 months post-discharge as the exposure window [14], with the baseline date set at the end of this period. This window was divided into four 3-month time windows (0–3, 3–6, 6–9, and 9–12 months). Patients with ≥1 BP measurements in ≥three of the four aforementioned time windows were included in the subsequent analysis (**Figure 1**). The group-based trajectory model (GBTM) was used to identify distinct long-term patterns of SBP levels. GBTM is an emerging semi-parametric statistical method that identifies heterogeneity in longitudinal data by classifying individuals into latent groups (clusters) with similar trajectories of SBP over the exposure window [15]. Accordingly, in this study, the long-term SBP groups were primarily defined based on the latent clusters identified through GBTM. Grouped SBP levels were defined as the primary exposure, while long-term DBP, mid-BP (calculated as (SBP+DBP) / 2), and mean arterial BP (calculated as ((1 / 3) × SBP + (2 / 3) × DBP)) levels were analysed as secondary exposures [16].

### Outcome definition

We considered a 36-month follow-up period following the exposure window as the principal analysis period (**Figure 1**) [17]. The primary outcomes of this study included recurrent stroke (ICD-10 = I60–I64) and MACE (including recurrent stroke, ischemic heart disease (IHD = I20–I25), and heart failure (I11.0, I13.0, I13.2, I50, I50.1, and I50.9); Table S1 in the **Online Supplementary Document**) that occurred in the principal analysis period. All these outcomes were tracked and identified from the inpatient medical records or death registration records by ICD-10 codes. Detailed descriptions of the outcomes can be found in Supplementary methods in the **Online Supplementary Document**. Patients were followed up from the baseline date until either recurrent stroke, MACE, death, the end of a 36-month follow-up period, or 1 October 2023, whichever came first.

### Covariates

Each patient's baseline characteristics at or before the baseline were analysed, including demographic information (age, sex, ethnic groups, marital status, education levels, , and insurance payment), lifestyle (smoking and alcohol consumption status which are retrieved from chronic disease management records), subcategories of current stroke (lacunar cerebral infarction (I63.801) and non-lacunar cerebral infarction), comorbidities (hypertension, diabetes, hyperlipidaemia, IHD, heart failure, atrial fibrillation, carotid artery stenosis, chronic kidney disease (CKD), hemiplegia, transient ischemic attack (TIA), and previous stroke), and therapies received (antihypertensive drugs, antidiabetic drugs, statins, endovascular therapy, thrombolysis, anticoagulant therapy, and antiplatelet therapy). Detailed definition of covariates and methods for addressing missing values can be found in Supplementary methods and Table S1 in the **Online Supplementary Document**.

### Statistical analysis

We initially fitted GBTMs with one to seven trajectory groups in cubic form for SBP. Subsequently, the optimal model was identified based on the lowest Akaike information criterion (AIC) and Bayesian information criterion (BIC), under the constraint that the smallest group comprised more than 5% of the sample [18]. To reduce misclassification, we further required an average posterior probability ≥0.70 for each group [8].

The propensity score overlap weighting (PSOW) method was used to balance baseline characteristics among different SBP level groups, considering all covariates (demographics, lifestyle, stroke subtypes, comorbidities, and therapies) [19]. We assessed balance across different SBP groups using the standardised mean difference (SMD) before and after PSOW. Groups were considered comparable if SMD was <0.100 [19].

We calculated the incidence rates (IRs; per 1000 person-years) and compared the survival curves of different SBP levels using the Kaplan-Meier method with a Log-rank test. The proportional hazards assumption was verified using Schoenfeld residuals, with no significant violations detected (*P* = 0.110). Subsequently, a set of Cox regression models, including three conventional models and one PSOW-weighted model, was used to evaluate the association between long-term SBP levels and cardiovascular outcomes, and to estimate the corresponding hazard ratios (HRs) and 95% confidence intervals (CIs). To quantitatively assess the health benefits gained from optimal SBP levels, we calculated the age-scaled restricted mean survival time (RMST), which was defined as the area under the survival curve up to a predefined time point [20]. Differences in RMST between groups can be interpreted as the years of event-free survival gained or lost due to specific SBP levels across age groups [21]. We reported the RMST within the age range of 45 to 80 years. Detailed descriptions of Cox regression models and RMST can be found in the Supplementary methods in the **Online Supplementary Document**.

We conducted subgroup analyses stratified by current stroke subtypes (lacunar cerebral infarction/non-lacunar cerebral infarction), age groups (<70/≥70 years old), and sex (women/men). Statistical significance of interactions between SBP levels and current stroke subtypes, age groups, and sex was examined using likelihood-ratio tests, respectively. Several sensitivity analyses were also conducted. For example:

1) non-stroke and non-MACE-related deaths were treated as competing events using the Fine-Gray model,

2) hospital level (tertiary, secondary, or community primary) was included as a random effect in the Cox model to minimise the potential impact of variability in BP measurements across different medical settings, and

3) excluding patients whose posterior probability of assignment to their most likely SBP level group was <0.70, in order to reduce misclassification of SBP level group.

Detailed descriptions of additional sensitivity analyses are provided in Supplementary methods in the **Online Supplementary Document**. Moreover, we conducted several additional analyses to further investigate the relationship between SBP levels and subcategories of MACE (*e.g*. IHD and heart failure) and haemorrhagic transformation, which is a severe complication that includes all types of post-ischemic haemorrhages and significantly worsens the prognosis for IS survivors (Table S1 in the **Online Supplementary Document**) [22], as well as the relationship between secondary exposures and primary outcomes.

We examined the potential selection bias resulting from sample exclusion and assessed the sample size for the present study, with findings indicated that selection bias might not be a major concern (Supplementary methods and Figure S2 in the **Online Supplementary Document**) and that the sample size was sufficiently adequate. Detailed methods are provided in Supplementary methods in the **Online Supplementary Document**. All the analyses were performed utilising *R*, Version 4.1.2 (R Foundation for Statistical Computing, Vienna, Austria). Statistical tests followed a two-sided approach, and statistical significance was determined by considering a *P*-value <0.05.

## RESULTS

### Long-term SBP levels and patient characteristics

The GBTM analysis indicated a monotonic decrease in both the AIC and the BIC as the number of trajectory groups increased. However, models with four or more groups resulted in at least one group with a size smaller than 5% of the total sample. Consequently, the three-group model for SBP, in which the average posterior probability of assignment exceeded 0.70 for each group, was identified as the optimal model (Table S2 in the **Online Supplementary Document**). In the optimal three-group model, the groups were classified as ‘normal SBP,’ ‘high-normal SBP,’ and ‘uncontrolled SBP’ based on their relatively stable trajectories during the exposure window and their clinical relevance. During the exposure window, the normal, high-normal, and uncontrolled SBP groups exhibited mean SBP levels of 120 ± 10 mm Hg, 132 ± 9 mm Hg, and 146 ± 14 mm Hg, respectively (**Figure 1**; Table S3 in the **Online Supplementary Document**).

Among 11 357 eligible patients, those with ≥5 BP measurements were predominant (69.5%), with a majority being men (61.1%) and a mean age of 64.0 years (standard deviation (SD) = 10.8) (**Table 1**). There were 2120 patients in the normal SBP level group (18.7%), 7949 in the high-normal SBP level group (70.0%), and 1288 in the uncontrolled SBP level group (11.3%). Compared with the uncontrolled SBP level, patients with normal or high-normal SBP levels tended to be younger, have a lower proportion of widowhood or being single, junior high school education or below, workers or farmers, urban and rural resident medical insurance, current smokers, and current alcohol drinkers. In addition, they tended to have a lower burden of comorbidities, such as hypertension, CKD, TIA, and previous stroke. But they were more likely to receive antidiabetic drugs, statins, and antiplatelet medications. The SMD among the three SBP level groups ranged from 0.018 to 0.393. After PSOW, these groups tended to be comparable (all SMDs <0.100) (**Table 1**).

**Table 1:** Baseline characteristics of study population

	Overall (n = 11 357)	Normal SBP (n = 2120)*	High-normal SBP (n = 7949)*	Uncontrolled SBP (n = 1288)*	SMD before PSOW	SMD after PSOW
**Number of BP measurements, n (%)**						
3	1370 (12.1)	212 (10.0)	1084 (13.6)	74 (5.7)	-	-
4	2099 (18.5)	346 (16.3)	1603 (20.2)	150 (11.6)	-	-
≥5	7888 (69.5)	1562 (73.7)	5262 (66.2)	1064 (82.6)	-	-
**Demographics, n (%)**						
Men	6942 (61.1)	1343 (63.3)	4827 (60.7)	772 (59.9)	0.047	0.010
Age in years†	64.0 (10.8)	62.3 (10.3)	64.3 (10.9)	64.8 (10.7)	0.156	0.008
**Ethnic groups, n (%)**					0.033	0.010
Han	11175 (98.4)	2083 (98.3)	7826 (98.5)	1266 (98.3)		
Minority	5 (0.04)	2 (0.1)	3 (0.04)	0 (0.0)		
Unknown	177 (1.6)	35 (1.7)	120 (1.5)	22 (1.7)		
**Marital status, n (%)**					0.040	0.028
Married/remarried	10495 (92.4)	1953 (92.1)	7357 (92.6)	1185 (92.0)		
Divorced	229 (2.0)	54 (2.5)	149 (1.9)	26 (2.0)		
Widowed	474 (4.2)	82 (3.9)	335 (4.2)	57 (4.4)		
Single	99 (0.9)	19 (0.9)	67 (0.8)	13 (1.0)		
Unknown	60 (0.5)	12 (0.6)	41 (0.5)	7 (0.5)		
**Education levels, n (%)**					0.191	0.019
Junior high school or below	6231 (54.9)	1062 (50.1)	4371 (55.0)	798 (62.0)		
Senior high school or vocational school	2748 (24.2)	562 (26.5)	1903 (23.9)	283 (22.0)		
College or above	1343 (11.8)	315 (14.9)	928 (11.7)	100 (7.8)		
Unknown	1035 (9.1)	181 (8.5)	747 (9.4)	107 (8.3)		
**Jobs, n (%)**					0.162	0.040
Civil servants/managers/professionals	364 (3.2)	93 (4.4)	250 (3.1)	21 (1.6)		
Office workers	794 (7.0)	170 (8.0)	550 (6.9)	74 (5.7)		
Business and service workers	445 (3.9)	90 (4.2)	310 (3.9)	45 (3.5)		
Workers/farmers	1944 (17.1)	362 (17.1)	1342 (16.9)	240 (18.6)		
Retirees	4137 (36.4)	763 (36.0)	2914 (36.7)	460 (35.7)		
Unemployed	1659 (14.6)	272 (12.8)	1194 (15.0)	193 (15.0)		
Others	2014 (17.7)	370 (17.5)	1389 (17.5)	255 (19.8)		
**Insurance payment, n (%)**					0.155	0.021
Employee medical insurance	5018 (44.2)	1051 (49.6)	3472 (43.7)	495 (38.4)		
Urban and rural resident medical insurance	2481 (21.8)	409 (19.3)	1780 (22.4)	292 (22.7)		
Others	3858 (34.0)	660 (31.1)	2697 (33.9)	501 (38.9)		
**Current smoker, n (%)**					0.099	0.018
Yes	1620 (14.3)	313 (14 .8)	1106 (13.9)	201 (15.6)		
No	9394 (82.7)	1726 (81.4)	6616 (83.2)	1052 (81.7)		
Unknown	343 (3.0)	81 (3.8)	227 (2.9)	35 (2.7)		
**Current alcohol drinker, n (%)**					0.090	0.008
Yes	1719 (15.1)	334 (15.8)	1157 (14.6)	228 (17.7)		
No	8821 (77.7)	1637 (77.2)	6202 (78.0)	982 (76.2)		
Unknown	817 (7.2)	149 (7.0)	590 (7.4)	78 (6.1)		
**Subcategories of current stroke, n (%)**					0.036	0.020
Lacunar cerebral infarction	4469 (39.4)	793 (37.4)	3181 (40.0)	495 (38.4)		
Non-lacunar cerebral infarction	6888 (60.6)	1327 (62.6)	4768 (60.0)	793 (61.6)		
**Comorbidities, n (%)**						
Hypertension	9319 (82.1)	1469 (69.3)	6669 (83.9)	1181 (91.7)	0.393	0.034
Diabetes	5564 (49.0)	1237 (58.3)	3767 (47.4)	560 (43.5)	0.200	0.026
Hyperlipidaemia	813 (7.2)	175 (8.3)	538 (6.8)	100 (7.8)	0.038	0.007
IHD	1590 (14.0)	340 (16.0)	1088 (13.7)	162 (12.6)	0.066	0.014
Heart failure	537 (4.7)	119 (5.6)	356 (4.5)	62 (4.8)	0.035	0.005
Atrial fibrillation	183 (1.6)	69 (3.3)	98 (1.2)	16 (1.2)	0.091	0.005
Carotid artery stenosis	357 (3.1)	90 (4.2)	236 (3.0)	31 (2.4)	0.069	0.006
CKD	575 (5.1)	98 (4.6)	379 (4.8)	98 (7.6)	0.083	0.014
Hemiplegia	205 (1.8)	49 (2.3)	140 (1.8)	16 (1.2)	0.054	0.012
TIA	219 (1.9)	36 (1.7)	156 (2.0)	27 (2.1)	0.019	0.025
Previous stroke	2199 (19.4)	439 (20.7)	1470 (18.5)	290 (22.5)	0.067	0.007
**Therapy, n (%)**						
Antihypertensive drugs	8064 (71.0)	1349 (63.6)	5734 (72.1)	981 (76.2)	0.184	0.048
Antidiabetic drugs	4925 (43.4)	1090 (51.4)	3355 (42.2)	480 (37.3)	0.191	0.015
Statins	7065 (62.2)	1457 (68.7)	4858 (61.1)	750 (58.2)	0.146	0.015
Endovascular therapy	74 (0.7)	27 (1.3)	41 (0.5)	6 (0.5)	0.058	0.001
Intravenous thrombolysis	135 (1.2)	30 (1.4)	88 (1.1)	17 (1.3)	0.018	0.007
Anticoagulant therapy	1009 (8.9)	256 (12.1)	641 (8.1)	112 (8.7)	0.089	0.012
Antiplatelet therapy	6500 (57.2)	1288 (60.8)	4514 (56.8)	698 (54.2)	0.089	0.027

BP – blood pressure, CKD – chronic kidney disease, IHD – ischemic heart disease, PSOW – propensity score overlap weighting, SBP – systolic blood pressure, SMD – standardised mean difference, TIA – transient ischemic attack

*Long-term SBP groups were constructed based on the latent clusters identified using the group-based trajectory model

†Continuous variables are presented as mean (standard deviation).

### Association of SBP levels with the incident risks of cardiovascular outcomes

During a median follow-up period of 1.75 years (interquartile range (IQR) = 0.79–2.97 years), we observed a total of 202 (IR = 56.5 per 1000 person-years; 95% CI = 49.0–64.9) recurrent stroke cases in the normal SBP level group, 862 (IR = 60.7 per 1000 person-years; 95% CI = 56.7–64.9) cases in the high-normal SBP level group, and 160 (IR = 73.0 per 1000 person-years; 95% CI = 62.1–85.2) cases in the uncontrolled SBP level group. For MACE cases, the corresponding numbers were 391, 1746, and 284 in these three groups (**Table 2**). Among all MACE cases, IHD accounted for 42.4%, heart failure for 37.7%, and stroke for 19.9% (Figure S3 in the **Online Supplementary Document**).

**Table 2:** Association between long-term SBP levels and clinical outcomes in a 36-mo follow-up period

	Normal SBP (n = 2120)*	High-normal SBP (n = 7949)*	Uncontrolled SBP (n = 1288)*
**Recurrent stroke**			
Number of events	202	862	160
Incidence rate (95% CI)†	56.5 (49.0–64.9)	60.7 (56.7–64.9)	73.0 (62.1–85.2)
Crude model, HR (95% CI)‡	0.77 (0.63–0.95)§	0.84 (0.71–0.99)§	Ref.
Adjusted model 1, HR (95% CI)‡	0.79 (0.64–0.97)§	0.81 (0.68–0.96)§	Ref.
Adjusted model 2, HR (95% CI)‡	0.70 (0.57–0.87)§	0.77 (0.65–0.92)§	Ref.
Weighted model, HR (95% CI)‡	0.68 (0.54–0.86)§	0.78 (0.65–0.94)§	Ref.
**MACE**			
Number of events	391	1746	284
Incidence rate (95% CI)†	118.4 (107.0–130.8)	135.5 (129.2–142.0)	140.8 (124.9–158.2)
Crude model, HR (95% CI)‡	0.84 (0.72–0.97)§	0.97 (0.85–1.10)	Ref.
Adjusted model 1, HR (95% CI)‡	0.87 (0.75–1.01)	0.94 (0.83–1.07)	Ref.
Adjusted model 2, HR (95% CI)‡	0.73 (0.63–0.86)§	0.88 (0.78–1.00)§	Ref.
Weighted model, HR (95% CI)‡	0.76 (0.64–0.90)§	0.89 (0.78–1.02)	Ref.

CI – confidence interval, HR – hazard ratio, MACE – major adverse cardiovascular events, SBP – systolic blood pressure

*Long-term SBP groups were constructed based on the latent clusters identified using the group-based trajectory model.

†Unit of incidence rate: /1000 person-years.

‡Crude model: without any adjustment. Adjusted model 1: adjusted for age and sex. Adjusted model 2: adjusted for sex, age, ethnic groups, marital status, education levels, jobs, insurance payment, smoking, alcohol drinking, hypertension, diabetes, hyperlipidaemia, atrial fibrillation, carotid artery stenosis, chronic kidney disease, hemiplegia, transient ischemic attack, previous stroke, antihypertensive drugs, antidiabetic drugs, statins, endovascular therapy, thrombolysis, anticoagulant therapy, and antiplatelet therapy. Weighted model, weighted by propensity score overlap weights.

§*P* < 0.05 was considered statistically significant.

The results from different models indicated consistent findings. Specifically, in the weighted models, the normal and high-normal SBP levels were associated with a 32% (weighted HR = 0.68; 95% CI = 0.54–0.86) and a 22% (weighted HR = 0.78; 95% CI = 0.65–0.94) lower risk of recurrent stroke, respectively, compared to the uncontrolled SBP level. Furthermore, the normal SBP level was also associated with a 24% (weighted HR = 0.76; 95% CI = 0.64–0.90) lower incident risk of MACE, when compared to the uncontrolled SBP level. However, the high-normal SBP level exhibited a non-significant trend toward reduced incident risk of MACE (weighted HR = 0.89; 95% CI = 0.78–1.02) (**Table 2**). Additionally, the Kaplan-Meier curves comparing the survival probability among three SBP level groups were appeared to be consistent with the primary findings (Figure S4 in the **Online Supplementary Document**).

The results of the standalone comparison of recurrent stroke and MACE risk between normal and high-normal SBP levels are provided in Supplementary results and Table S4 in the **Online Supplementary Document**.

### Event-free survival time gained from optimal SBP levels

**Figure 2:**
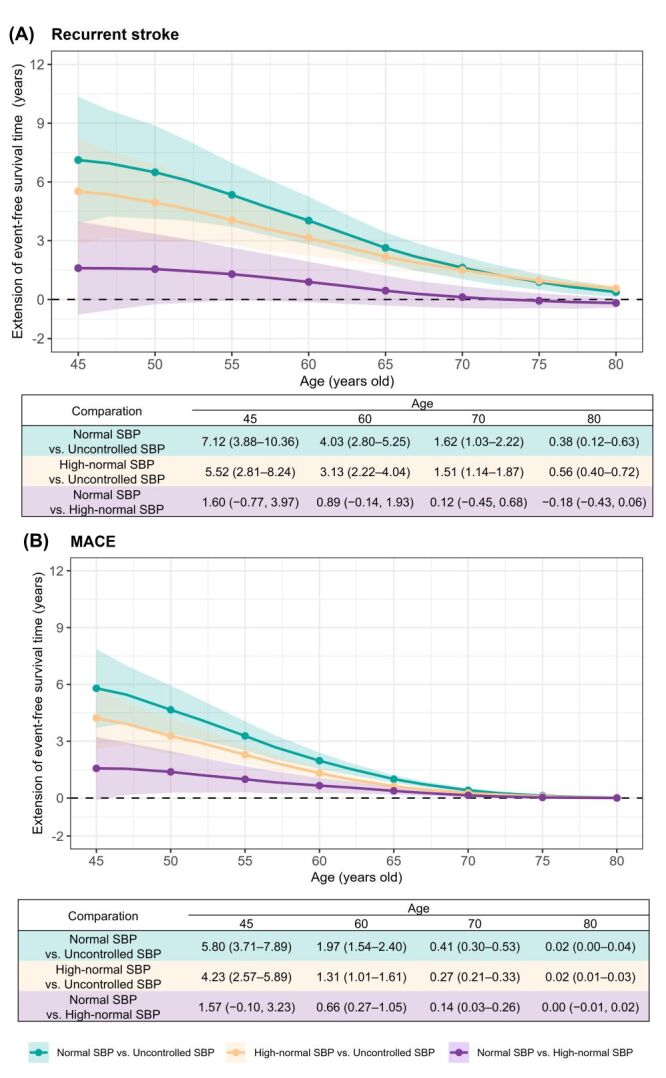
Extension of event-free survival time gained from long-term normal or high-normal SBP levels by age for recurrent stroke and MACE. Long-term SBP groups were constructed based on the latent clusters identified using the group-based trajectory model. MACE – major adverse cardiovascular events, SBP – systolic blood pressure.

Compared to the uncontrolled SBP level, event-free survival time for recurrent stroke and MACE gained from the normal and high-normal SBP levels decreased with increasing age. Specifically, in the normal SBP level group, IS patients aged 45 to 70 years gained event-free survival time ranging from 7.12 to 1.62 years for recurrent stroke and from 5.80 to 0.41 years for MACE. Additionally, in the high-normal SBP level group, patients in the same age bracket gained event-free survival time ranging from 5.52 to 1.51 years for recurrent stroke and from 4.23 to 0.27 years for MACE. For patients aged 70 to 80 years, the event-free survival time for recurrent stroke and MACE obtained from either the normal SBP or the high-normal SBP levels were significantly reduced and gradually approached zero, ranging from 1.62 to 0.02 years. In terms of the magnitude of these benefits, within the age range of 45 to 70 years, the event-free survival time gained by the normal SBP level appears to be greater than that gained by the high-normal SBP level. However, after the age of 70, the event-free survival time obtained from both levels tended not to be significantly different. When comparing the normal SBP level to the high-normal SBP level (used as a reference) separately, the results were consistent with the above findings (**Figure 2**; Table S5 in the **Online Supplementary Document**).

### Subgroup, sensitivity, and additional analyses

**Figure 3:**
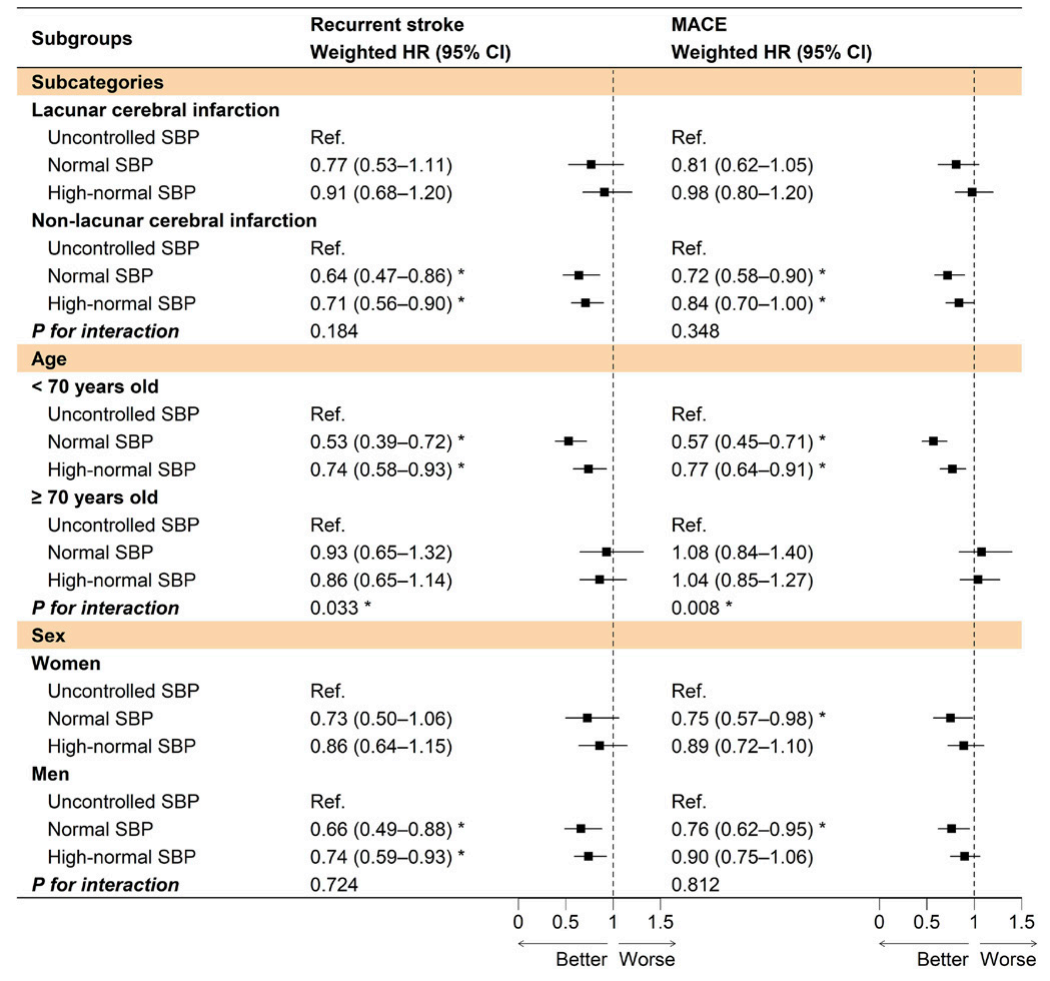
Subgroup analysis of the risks of stroke recurrent and MACE associated with long-term SBP levels within a 36-month follow-up period. Long-term SBP groups were constructed based on the latent clusters identified using the group-based trajectory model. Weighted HRs were estimated by propensity score-overlap weighted Cox regression models. *P < 0.05 was considered statistically significant. 95% CI – 95% confidence interval, HR – hazard ratio, MACE – major adverse cardiovascular events, SBP – systolic blood pressure.

In subgroup analyses, consistent risk reduction effects of normal or high-normal SBP levels on the recurrent stroke and MACE were observed across most subgroups. It is worth noting that among patients aged <70 years old, the associations of normal or high-normal SBP levels with lower risks of recurrent stroke and MACE may be more pronounced compared to those who were ≥70 years old (*P _interactions_*<0.05) (**Figure 3**). The favourable associations between the normal or high-normal SBP levels and lower risks of recurrent stroke and MACE remained largely unchanged across various sensitivity analyses (Supplementary results and Table S6 in the **Online Supplementary Document**). Additional analyses results can be found in Supplementary results, Figure S5, and Table S7–S10 in the **Online Supplementary Document**.

## DISCUSSION

In this real-world cohort study, we identified that IS patients with the long-term normal or high-normal SBP were associated with lower risks of recurrent stroke or MACE, and potentially gained longer event-free survival time, compared to those with the long-term uncontrolled SBP. However, the magnitude of this health benefit would decrease with age.

BP management plays a crucial role in the prognosis of stroke. However, reaching a definitive consensus on the optimal target for BP remains elusive. A BP target of <130 / 80 mm Hg is widely accepted [23], but a lower BP target has not yet been recommended by current clinical guidelines. In our study, we found that maintaining a long-term sustained normal or lower SBP level (x̄ = 120 ± 10 mm Hg) might be more effective in reducing the risk of adverse cardiovascular events in IS patients. These findings have not been previously reported in similar studies [14,18]. In a post-hoc analysis of the China Antihypertensive Trial in Acute Ischemic Stroke, Zheng et al. [14] identified four distinct SBP trajectories among 3479 patients with IS (age >22 years). However, the mean SBP of all four trajectories exceeded 130 mm Hg, precluding further evaluation of the impact of a lower SBP level (<130 mm Hg) on patient prognoses. In a cohort study by the Clinical Research Collaboration for Stroke in Korea (mean age = 67 ± 13 years), a one-year SBP trajectory with a mean of 114 mm Hg was identified. However, the sample size (n = 5514) was relatively small and the follow-up period was one year, leading to a statistically non-significant association between the SBP trajectory and the risk of adverse cardiovascular outcomes (adjusted HR = 0.86; 95% CI = 0.71–1.04) [18]. Furthermore, several randomised controlled trials (RCTs) have evaluated BP interventions for secondary prevention in patients with cerebrovascular disease [24,25]. However, these studies encompassed heterogeneous patient populations, and the majority of patients in most studies did not achieve SBP targets below 120 mm Hg. Therefore, conclusive evidence regarding the protective impact of maintaining SBP less than 120 mm Hg on the prognosis of IS patients remains limited. Recently, an RCT conducted across 116 clinical centres in China (n = 11 255) demonstrated that intensive BP control (SBP<120 mm Hg) was more effective than standard BP control (SBP<140 mm Hg) in reducing the risk of major vascular events among hypertension patients with high cardiovascular risk [26]. The HR for intensive BP control *vs*. standard BP control was 0.88 (95% CI = 0.72–1.07) in the subgroup of patients with a history of stroke. Although the HR did not reach statistical significance, the trend toward a protective effect suggested potential benefits of intensive BP control in secondary stroke prevention. Thus, our findings may add real-world evidence supporting the need for other RCTs to examine and confirm the potential benefits of intensive BP control on the prognosis of patients with IS.

In the present study, we observed that IS patients with normal or high-normal SBP may derive greater health benefits compared to those with uncontrolled SBP, especially among younger IS patients. The findings supported that the earlier and long-term hypertension management was crucial for IS secondary prevention. However, after the age of 70, the event-free survival times obtained from the normal and high-normal SBP tended not to be significantly different. This result is consistent with the findings stratified by age (<70 /≥70) in our study and the findings of Zhang et al. [27]. They observed that intensive treatment (SBP = 110 to 130 mm Hg) had a significant cardiovascular protective effect in individuals aged <70 years (HR = 0.75; 95% CI = 0.58–0.98), but this effect was no longer significant in those aged ≥70 years (HR = 0.73; 95% CI = 0.50–1.05). From the perspective of BP management, maintaining a lower BP target requires more intensive antihypertensive drug therapy, entails greater risks of side effects, and involves higher medication costs [28,29]. Moreover, not all older patients can achieve the goal of intensive BP lowering. The results from an RCT indicated that only 32.0% of patients with IS were able to reach the goal of intensive BP lowering [30]. Therefore, recommendations for appropriate targets of long-term BP management should take patient's age characteristics into account.

This study has several strengths. First, the use of health information database provides a large real-world sample size, which enhances the statistical power of our analysis. Second, we included more detailed disease history and treatment information for IS patients, which allows for robust control of confounding factors. Third, we calculated the event-free survival time to estimate the health benefits potentially gained from optimal SBP levels, which were not reported in previous similar studies. Finally, we can obtain multiple BP measurement records from chronic disease management records, inpatient or outpatient settings, better reflecting the actual BP changes within the study population. However, this study also has several limitations. First, due to a lack of data availability, additional clinical dimensions, such as radiological features (*e.g*. infarction locations and areas) and biochemical indicators (*e.g*. fasting lipid and glucose levels), were not considered in the present study. Second, although we had adopted measures, standardising BP measurements remains challenge due to potential variations in measurement protocols across different medical settings, which may introduce bias and marginally distort the observed associations in either direction. Third, outcome events were ascertained from inpatient or death records, while outpatient diagnoses were not included. Consequently, the actual incidence of recurrent stroke and MACE along with the potential beneficial effect of the conclusions might be slightly underestimated. Fourth, despite employing the PSOW method, residual confounding was still possible. Meanwhile, misestimation of results could occur due to potential immortal time bias [31]. Finally, the findings were based solely on the population in Shenzhen, China. Caution should be exercised when extrapolating these results to other regions or countries. Therefore, more in-depth and well-designed research will be necessary in the future.

## CONCLUSIONS

Our findings indicated that maintaining a normal or high-normal long-term SBP level for IS patients may be associated with reduced risks of adverse cardiovascular events and potentially yield greater health benefits.

## Additional material


Online Supplementary Document
