# Supplementary material for: Long-term systolic blood pressure and cardiovascular risks among patients with ischemic stroke: a register-based cohort study
Source: J Glob Health. 2025 Nov 7;15:04321. doi: 10.7189/jogh.15.04321 (PMC12593165; doi:10.7189/jogh.15.04321)
Supplement: Online Supplementary Document [file jogh-15-04321-s001.pdf]

Supplement to: Mo C, Li X, Wang S, Wang J, He L, Peng R, Zheng J, Liang F, Gu D. Long-term systolic blood pressure and cardiovascular risks among patients with ischemic stroke: a register-based cohort study. J Glob Health. 2025;15:04321.

Supplementary methods **1**

Detailed descriptions of outcomes 1

Methods of addressing missing data 1

Cox regression analysis procedure 1

Estimation of restricted mean survival time 2

Detailed description of sensitivity analyses 2

Examination of potential selection bias 3

Sample size assessment 3

Supplementary results **5**

Result of standalone comparison (normal vs. high-normal SBP levels) 5

Results of sensitivity analyses 5

Additional analyses results 6

Association of SBP levels with the incident risks of subcategories of MACE 6

Description of the long-term levels of other BP indicators 6

Association of other BP levels with the incident risks of cardiovascular outcomes 7

Supplementary figures **8**

Figure S1. Flow chart of present study 8

Figure S2. Standardized mean difference between the analysis samples before (N=15 772) and after (N= 11 357) exclusion 9

Figure S3. Proportion of subtypes for MACE (N= 2421) 10

Figure S4. Kaplan-Meier curves for survival probability 11

Figure S5. Predicted long-term levels for DBP, mid-blood BP, and mean arterial BP 12

Supplementary tables **13**

Table S1. Definition of variables 13

Table S2. Parameters of four blood pressure long-term level patterns 16

Table S3. Values of each distinct long-term levels of blood pressure in four measurements 18

Table S4. Risk of clinical outcomes in long-term normal vs. high-normal SBP levels within a 36-month follow-up period 20

Table S5. Restricted mean survival time of different long-term SBP levels by age for recurrent stroke and MACE 21

Table S6. Sensitivity analysis of association between long-term SBP levels and the risk of recurrent stroke and MACE 23

Table S7. Association between long-term SBP levels and ischemic heart disease, heart failure, and hemorrhagic transformation during a 36-month follow-up period.................. 25

Table S8. Association between long-term DBP levels and clinical outcomes during a 36-month follow-up period 26

Table S9. Association between long-term mid-BP levels and clinical outcomes during a 36-month follow-up period 27

Table S10. Association between long-term mean arterial BP levels and clinical outcomes during a 36-month follow-up period 28

Supplementary methods

Detailed descriptions of outcomes

Recurrent stroke referred to a recurrence of any type of strokes for the ischemic stroke (IS) patients, including IS (International Classification of Diseases 10th Revision, ICD-10 code: I63), intracerebral hemorrhage (I61 and I62), subarachnoid hemorrhage (I60), and unspecified stroke (I64). Major cardiovascular events (MACE) were defined as a composite event that included recurrent stroke, ischemic heart disease (ICD-10 code: I20–I25), and heart failure (I11.0, I13.0, I13.2, I50, I50.1, I50.9; Supplementary Table S1). All these outcomes were tracked in a medical registry-based setting and were identified from the inpatient medical records or death registration records by ICD-10 codes. In cases where individuals died from recurrent stroke or MACE as the primary cause, it would also be classified as an event of recurrent stroke or MACE. However, hospitalizations for reasons such as contact with health-care institutions for examination or investigation (ICD-10 codes: Z00–Z13) and specialized procedures or prognostic care (Z40–Z54; Supplementary Table S1) should not be considered as outcomes of interest, even if these records included ICD-10 codes for stroke or MACE, because these hospitalizations were not due to the onset of diseases.

Methods of addressing missing data

The proportion of missing values among ethnic groups, marital status, education levels, current smoker, and current alcohol drinker were ≤9.1%, and missing values were classified separately as the "unknown" category in these covariates [1].

Cox regression analysis procedure

A set of Cox regression models were used to evaluate the association between long-term SBP levels and cardiovascular outcomes, and to estimate the corresponding hazard ratios and 95% confidence intervals. These models included: a crude model with no adjustment; Adjusted

Model 1 (adjusted for age and sex); Adjusted Model 2 (further adjusted for demographics, lifestyle factors, stroke subcategories, comorbidities, and therapies based on Model 1); and a weighted model using propensity score-overlap weight [2].

Estimation of restricted mean survival time

The restricted mean survival time (RMST) is defined as the area under the curve of the survival function up to τ ($< \infty$):

$$\mu_\tau = \int_0^\tau S(t)dt,$$

where $S(t)$ is the survival function of a time-to-event variable of interest. The interpretation of the RMST is that "when we follow up patients for τ, patients will survive for μ_τ on average," which is quite straightforward and clinically meaningful summary of the censored survival data [3].

In the present study, we computed the RMST using age as time scales, respectively. For the age-based method, the RMST at each year of age can be estimated as the area under the survival curve from a specific age up to a predefined age (or life expectancy) [4]. Subsequently, differences in the area under the survival curve (i.e., RMST) between patients in the different systolic blood pressure (SBP) level groups can be interpreted as mean years of life gained (or lost) due to a specific SBP level. However, it is important to highlight that a critical statistical assumption for generating reliable long-term survival projections from short-term follow-up data is that a patient's risk of an event depends only on age and intervention (i.e., SBP level in the present study), regardless of the duration of exposure to the intervention [5,6]. We reported the RMST within the age range of 45 to 80 years, with 45 years as the lower age limit of the study population and 80 years approximating China's life expectancy (77.7 years in 2019) [7].

Detailed description of sensitivity analyses

Several sensitivity analyses were performed: (1) we treated non-stroke or non-MACE-related deaths as competing events using the Fine-Gray model; (2) to minimize the potential impact of variability in BP measurements across different medical settings, we incorporated the hospital level as a random-effect term in the Cox models. Hospitals were classified into tertiary,

secondary, and community (primary) levels. For patients with BP measurements recorded at multiple hospitals during the exposure window, the hospital level with the highest recording frequency was assigned; (3) to reduce misclassification of SBP level group, we excluded patients whose posterior probability of assignment to their most likely group was <0.70—a threshold commonly used in group-based trajectory analyses [8]; (4) we excluded the patients with prevalent hemiplegia due to potential differences in BP measurements between hemiplegic and non-hemiplegic individuals; (5) we excluded the patients with previous stroke or transient ischemic attack; (6) we excluded the patients with less than four BP measurements; (7) we included only patients who had at least one BP measurement in all four measurement windows due to the potential impact of seasonal variations on BP fluctuations; (8) we used the inverse probability of treatment weighting method to balance baseline covariates among SBP levels groups [9]; and (9) we further adjusted the mean SBP within the exposure window and four measurement windows, respectively.

Examination of potential selection bias

To detect potential selection bias resulting from sample exclusion, we also calculated the standardized mean difference (SMD) between the analysis samples before (N=15 772) or after (N=11 357) exclusion. The results suggested that selection bias might not be a major concern as most SMD values were below 0.100 (Supplementary Figure S2).

Sample size assessment

In accordance with the methodology outlined by *Hsieh et al.*,[10] we utilized the "*survivalpwr*" package [11] to estimate the minimum sample size for this study. We estimated that a sample size of at least 6318 participants would provide 90% power at a two-sided $P=0.05$ to detect a 22% difference in the risk of the primary outcome between SBP level groups. Therefore, the sample size (N=11 357) of the present study was completely adequate.

References:

1 Li F, Zhu B, Liao J, Cheng Z, Jin C, Mo C, et al. Ambient Air Pollutants and Incident

Microvascular Disease: A Cohort Study. Environ Sci Technol. 2022;56:8485-8495.

2 Thomas LE, Li F, Pencina MJ. Overlap Weighting: A Propensity Score Method That Mimics Attributes of a Randomized Clinical Trial. JAMA. 2020;323:2417-2418.

3 Tian L, Zhao L, Wei LJ. Predicting the restricted mean event time with the subject's baseline covariates in survival analysis. Biostatistics. 2014;15:222-233.

4 Claggett B, Lachin JM, Hantel S, Fitchett D, Inzucchi SE, Woerle HJ, et al. Long-Term Benefit of Empagliflozin on Life Expectancy in Patients With Type 2 Diabetes Mellitus and Established Cardiovascular Disease. Circulation. 2018;138:1599-1601.

5 Claggett B, Packer M, McMurray JJ, Swedberg K, Rouleau J, Zile MR, et al. Estimating the Long-Term Treatment Benefits of Sacubitril-Valsartan. N Engl J Med. 2015;373:2289-2290.

6 Uno H, Claggett B, Tian L, Inoue E, Gallo P, Miyata T, et al. Moving Beyond the Hazard Ratio in Quantifying the Between-Group Difference in Survival Analysis. J Clin Oncol. 2014;32:2380-2385.

7 National Health Commission of the People's Republic of China. 2023 Annual Bulletin of Health and Family Planning Development in China. Available: http://www.nhc.gov.cn/cms-search/xxgk/getManuscriptXxgk.htm?id=6c037610b3a54f6c8535c515844fae96. 2024

8 Katsanos AH, Joundi RA, Palaiodimou L, Ahmed N, Kim JT, Goyal N, et al. Blood Pressure Trajectories and Outcomes After Endovascular Thrombectomy for Acute Ischemic Stroke. Hypertension. 2024;81:629-635.

9 Chesnaye NC, Stel VS, Tripepi G, Dekker FW, Fu EL, Zoccali C, et al. An introduction to inverse probability of treatment weighting in observational research. Clinical Kidney Journal. 2022;15:14-20.

10 Hsieh FY, Lavori PW. Sample-size calculations for the Cox proportional hazards regression model with nonbinary covariates. Control Clin Trials. 2000;21:552-560.

11 McGowan LD. survivalpwr: Power calculations for survival analyses [Z]. 2024.

Supplementary results

Result of standalone comparison (normal vs. high-normal SBP levels)

To further investigate whether there were differences in the risk of recurrent stroke and MACE between the normal and high-normal SBP levels, we conducted a standalone comparison specifically between these two groups. Using the high-normal SBP level as the reference, the normal SBP level showed a non-significant trend toward reduced recurrent stroke risk (weighted HR = 0.92; 95% CI = 0.79–1.08), while demonstrating a statistically significant reduction in MACE incidence (weighted HR = 0.86; 95% CI = 0.77–0.96; Supplementary Table S4).

Results of sensitivity analyses

The favorable associations between the normal or high-normal SBP levels and lower risks of recurrent stroke and MACE remained largely unchanged across various sensitivity analyses. Specifically, using uncontrolled SBP level serving as the reference and regarding the non-stroke or non-MACE-related deaths as competing events (Sensitivity analysis 1), the normal SBP level was associated with lower risks of recurrent stroke (weighted subdistribution hazard ratio[SHR] = 0.76; 95% CI = 0.61–0.95) and MACE (weighted SHR = 0.84; 95% CI = 0.72–0.97), respectively, while the high-normal SBP level was also associated with a reduced risk of recurrent stroke (weighted SHR = 0.82; 95% CI = 0.69–0.99). In Sensitivity analysis 2, among all BP measurements, 24.9% (2830), 37.1% (4218), and 37.9% (4309) were obtained from tertiary, secondary, and community (primary) hospitals, respectively. When hospital level (tertiary, secondary, or community) was included as a random effect in the Cox models, the results showed that the protective effect of a normal SBP level against recurrent stroke (weighted HR = 0.73; 95% CI = 0.58–0.92) and MACE (weighted HR = 0.83; 95% CI = 0.70–0.98) was statistically significant, while the protective effect of a high-normal SBP level against recurrent stroke was marginally significant (weighted HR = 0.78; 95% CI = 0.59–1.03). In Sensitivity analysis 3, a total of 231 (17.9%), 532 (25.1%), and 1097 (13.8%) patients

were excluded from the uncontrolled, normal, and high-normal SBP level groups, respectively. The sensitivity analysis demonstrated that, compared with uncontrolled SBP, normal SBP level was associated with lower risks of recurrent stroke (weighted HR = 0.61; 95% CI = 0.46–0.80) and MACE (weighted HR = 0.74; 95% CI = 0.61–0.90), while high-normal SBP level was also associated with a reduced risk of recurrent stroke (weighted HR = 0.78; 95% CI = 0.64–0.95). Moreover, the results of additional sensitivity analyses were largely consistent with the primary findings (Supplementary Table S6).

Additional analyses results

Association of SBP levels with the incident risks of subcategories of MACE

Compared to uncontrolled SBP level, the associations between normal or high-normal SBP levels with ischemic heart diseases risk were not statistically significant. However, normal SBP level was associated with a marginally significantly lower risk of incident heart failure (weighted HR = 0.68; 95% CI = 0.46–1.01). Moreover, both the normal and high-normal SBP levels were associated with a lower incident risk of hemorrhagic transformation, with the weighted HRs of 0.24 (95% CI = 0.10–0.55) and 0.44 (95% CI = 0.25–0.79), respectively, compared to the uncontrolled SBP level (Supplementary Table S7).

Description of the long-term levels of other BP indicators

The results from group-based trajectory models indicated that as the number of groups for diastolic blood pressure (DBP), mid-BP (computed as [SBP + DBP]/2), and mean arterial BP (calculated as [(1/3)×SBP + (2/3)×DBP]) levels increased from one to seven, both the AIC and the BIC values exhibited a consistent downward trend. However, when the number exceeded three, the proportion of the smallest group fell below 5%. Therefore, the best model for DBP, mid-BP, and mean arterial BP levels consisted of three groups, respectively (Supplementary Table S2).

For DBP long-term levels, we categorized them into “low-normal DBP”, “normal DBP”,

and “uncontrolled DBP” based on their temporal characteristics. Within the exposure window, these groups exhibited low to normal (mean 72 ± 7 mm Hg), normal (mean 81 ± 7 mm Hg), and relatively high (mean 90 ± 9 mm Hg) levels of DBP, respectively (Supplementary Figure S5 and Table S3).

For mid-BP long-term levels, we categorized them into “low-stable mid-BP”, “middle-stable mid-BP”, and “high-stable mid-BP” based on their temporal characteristics. Within the exposure window, these groups exhibited relatively low-stable (mean 99 ± 7 mm Hg), middle-stable (mean 107 ± 7 mm Hg), and high-stable (mean 118 ± 10 mm Hg) levels of mid-BP, respectively (Supplementary Figure S5 and Table S3).

For mean arterial BP long-term levels, we categorized them into “low-stable mean arterial BP”, “middle-stable mean arterial BP”, and “high-stable mean arterial BP” based on their temporal characteristics. Within the exposure window, these groups exhibited relatively low-stable (mean 91 ± 7 mm Hg), middle-stable (mean 99 ± 7 mm Hg), and high-stable (mean 109 ± 10 mm Hg) levels of mean arterial BP, respectively (Supplementary Figure S5 and Table S3).

Association of other BP levels with the incident risks of cardiovascular outcomes

Association analyses indicated that, compared with their respective uncontrolled or high-stable reference groups, (i) low-normal and normal DBP levels (vs. uncontrolled DBP level), (ii) low-stable and middle-stable mid-BP levels (vs. the high-stable mid-BP level), and (iii) low-stable and middle-stable mean arterial BP levels (vs. high-stable mean arterial BP level) were not significantly associated with lower risks of recurrent stroke or MACE (Supplementary Tables S8–S10).

Supplementary figures

Figure S1. Flow chart of present study

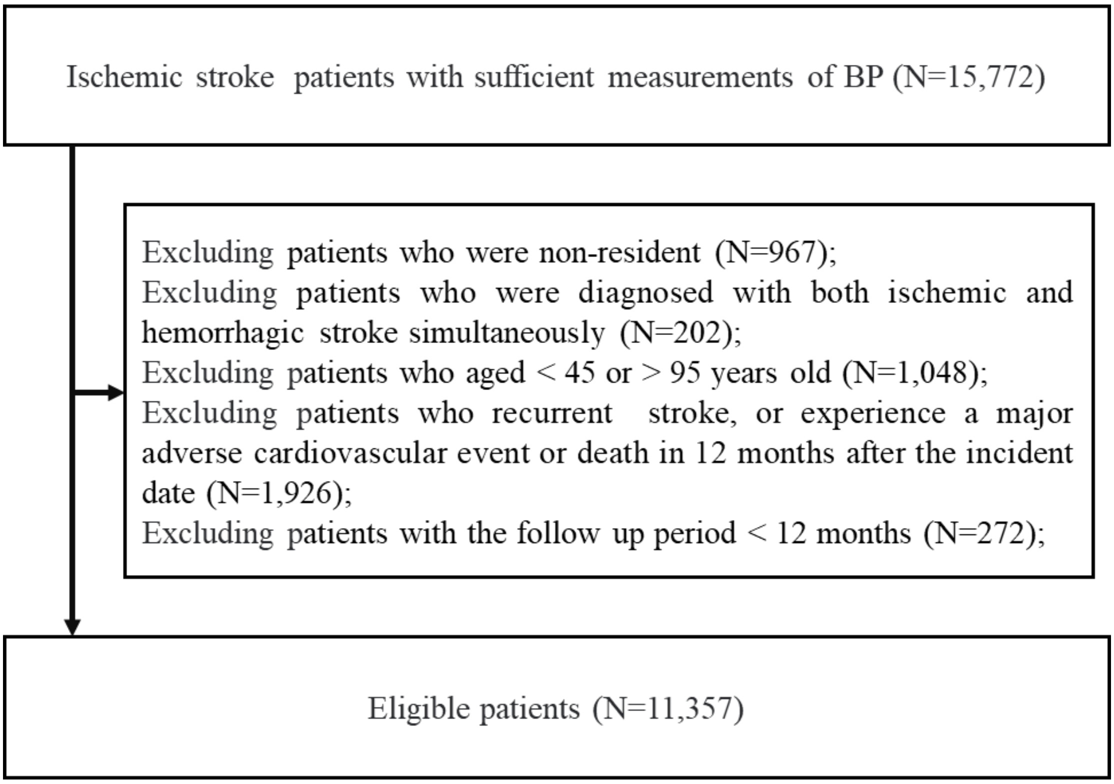

BP – blood pressure

Figure S2. Standardized mean difference between the analysis samples before (N=15 772) and after (N= 11 357) exclusion

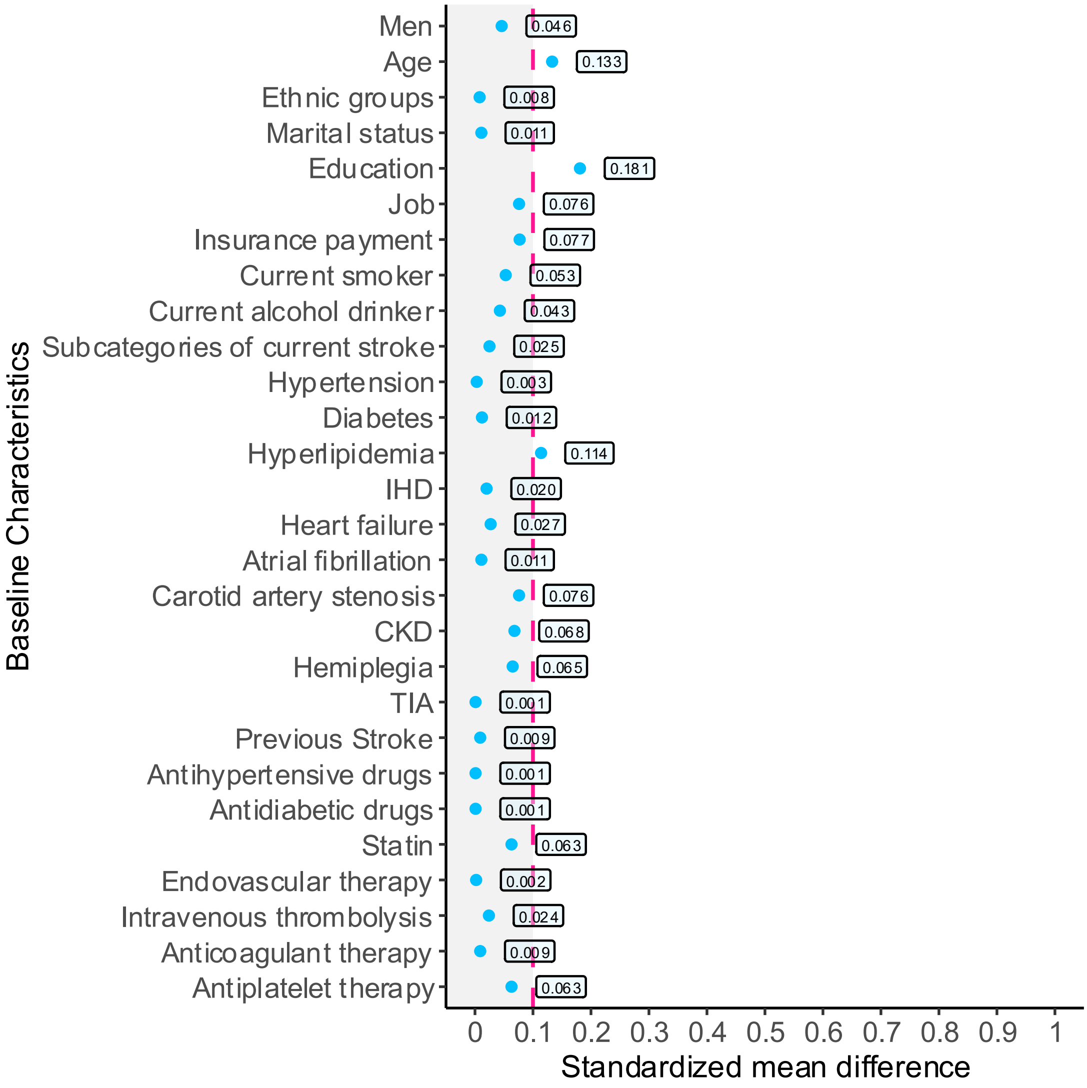

CKD – chronic kidney disease, IHD – ischemic heart disease, TIA – transient ischemic attack.

Figure S3. Proportion of subtypes for MACE (N= 2421)

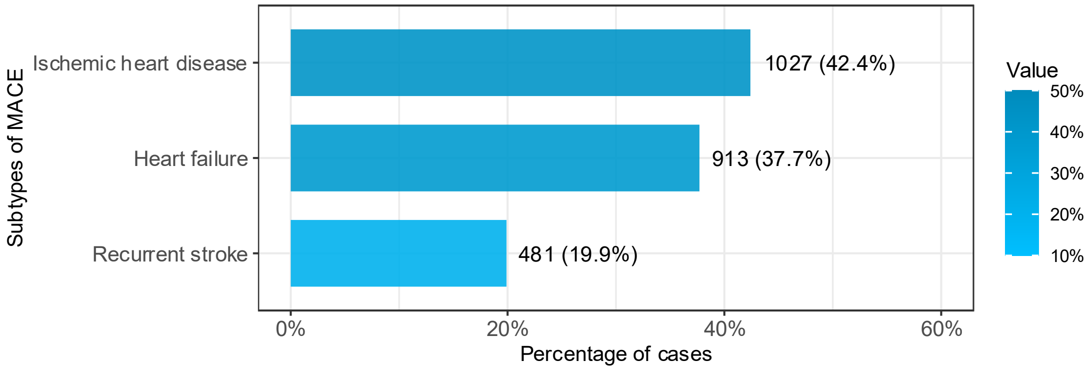

MACE – major cardiovascular events

Figure S4. Kaplan-Meier curves for survival probability

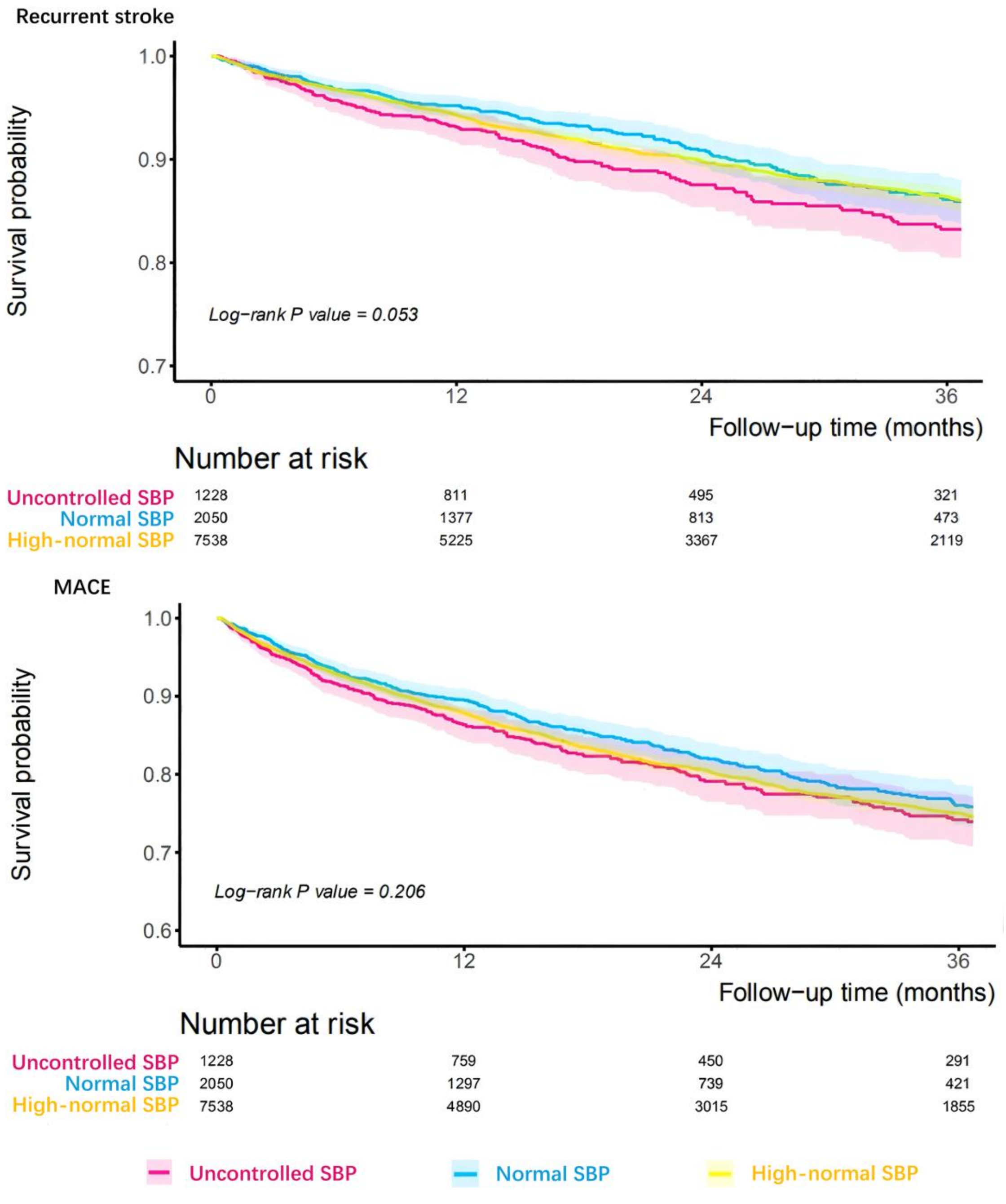

MACE – major cardiovascular events, SBP – systolic blood pressure

Long-term SBP groups were constructed based on the latent clusters identified using the group-based trajectory model

Figure S5. Predicted long-term levels for DBP, mid-blood BP, and mean arterial BP

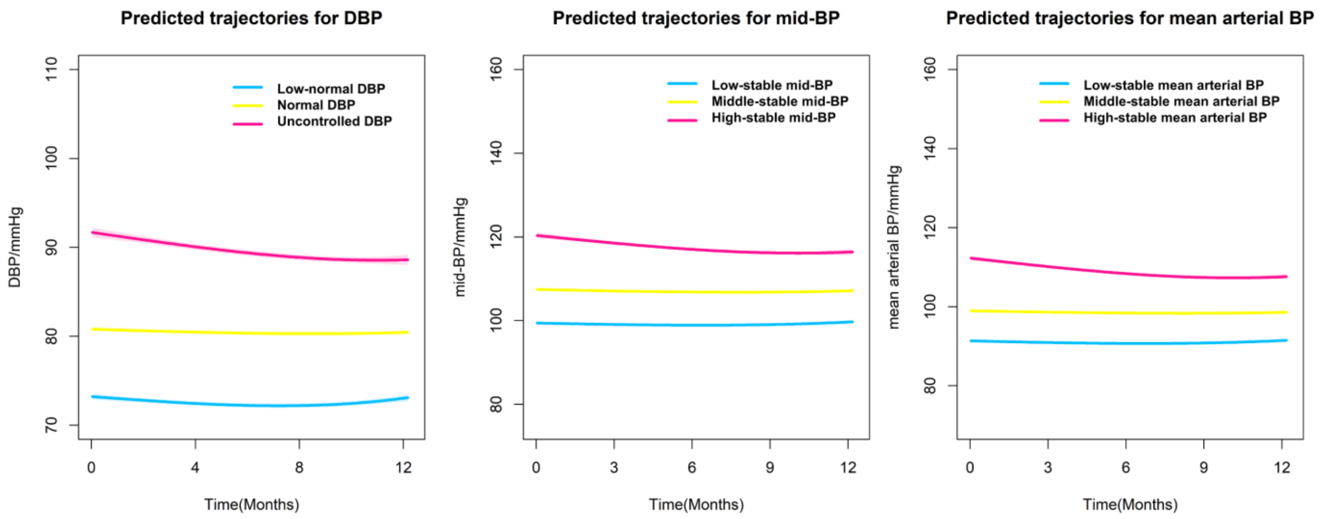

DBP – diastolic blood pressure, mid BP – mid-blood pressure, mean arterial BP – mean arterial blood pressure

Long-term BP groups were constructed based on the latent clusters identified using the group-based trajectory models

Supplementary tables

Table S1. Definition of variables

Variable	Definition
**Main diseases (ICD-10 codes)***	
Stroke	Ischemic stroke: I63;<br>-Lacunar cerebral infarction: I63.801;<br>-Non-lacunar cerebral infarction: Types of cerebral infarction other than lacunar infarction.<br>Intracerebral hemorrhage: I61, I62;<br>Subarachnoid hemorrhage: I60;<br>Unspecified stroke: I64;
Hypertension	Primary hypertension: I10;<br>Hypertensive heart disease: I11;<br>Hypertensive nephropathy: I12;<br>Hypertensive heart and renal disease I13;<br>Secondary hypertension: I15;
Diabetes	Type 1 diabetes: E10;<br>Type 2 diabetes: E11;<br>Malnutrition related diabetes: E12;<br>Other specified diabetes: E13;<br>Unspecified diabetes: E14;
Hyperlipidemia	Pure hypercholesterolemia: E78.0;<br>Pure hyperglyceridemia: E78.1;<br>Mixed hyperlipidemia: E78.2;<br>Hyperchylomicronemia: E78.3;<br>Other hyperlipidemia: E78.4;<br>Hyperlipidemia, unspecified: E78.5
Ischemic heart disease	Angina pectoris: I20<br>Acute myocardial infarction: I21<br>Subsequent myocardial infarction: I22<br>Certain current complications following acute myocardial infarction: I23<br>Other acute ischemic heart diseases: I24<br>Chronic ischemic heart disease: I25
Heart failure	Hypertensive heart disease with (congestive) heart failure: I11.0;<br>Hypertensive heart and kidney disease accompanied by (congestive) heart failure: I13.0;<br>Hypertensive heart and kidney disease are accompanied by (congestive) heart failure and

	kidney failure: I13.2;<br>Congestive Heart Failure: I50;<br>Left ventricular heart-failure: I50.1;<br>Unspecified heart failure: I50.9;
Atrial fibrillation	I48;
Carotid artery stenosis	I65.201–I65.203;<br>I65.206–I65.208;
Chronic kidney disease	Hypertensive renal failure: I12, I13;<br>Nephritic syndrome: N01, N03, N04, N05, N07, N08;<br>Chronic tubulo-interstitial nephritis: N11;<br>Chronic renal failure: N18, N19;<br>Renal tubular disease: N25;<br>Unspecified contracted kidney: N26;<br>Dialysis: Z49, Z99.2;
Hemiplegia	G80–G83;
Transient ischemic attack	G45.9;
Contact with the medical institutions	Z00–Z13: Contact with the medical institutions for examination and investigation;<br>Z40–Z54: Contact with the medical institutions for specialized operations and prognostic care;
Hemorrhagic transformation	Defined as a composite event of intracerebral hemorrhage (ICD-10 code: I61 and I62) and subarachnoid hemorrhage (I60) following discharge after ischemic stroke (I63).
**Therapy**	
Antihypertensive drugs	Treatment with antihypertensive drugs, including angiotensin-converting enzyme inhibitors (ACEI), angiotensin II receptor blockers (ARB), beta-blockers, calcium channel blockers (CCB), diuretics, alpha-blockers, and combination of antihypertensive medications.
Antidiabetic drugs	Treatment with antidiabetic drugs, including insulin, insulin analogs, GLP-1 receptor agonists, biguanides, sulfonylureas, thiazolidinediones, α-glucosidase inhibitors, dipeptidyl peptidase-IV (DPP-IV) inhibitors, sodium-glucose cotransporter 2 (SGLT2) inhibitors, and combination of antidiabetic medications.
Statins	Treatment with ss, such as Atorvastatin, Simvastatin, Rosuvastatin, Pravastatin, Lovastatin, Fluvastatin, and Pitavastatin.
Thrombectomy or embolectomy	The identification was made using the ICD-9-CM-3 code of 39.74.

Intravenous thrombolysis	Defined as treatment with thrombolytic drug, such as Rt-PA, Tenecteplase, and Urokinase.
Anticoagulant therapy	Defined as treatment with anticoagulant drug, such as Heparin, Warfarin, Rivaroxaban, Apixaban, Edoxaban, Bivalirudin, and Dabigatran Etexilate Capsules.
Antiplatelet therapy	Defined as treatment with thrombolytic drug, such as Aspirin, Clopidogrel, Dipyridamole, Ticagrelor, Cilostazol, and Indobufen.
Lifestyles	
Current smoker	Defined as self-reported current smoking.
Current alcohol drinker	Defined as self-reported daily or frequent alcohol consumption.

* Diseases were identified by using the codes of the International Classification of Diseases 10th Revision (ICD-10).

ICD-9-CM-3 – a standard system used to classify operations and medical procedures for billing purposes.

Table S2. Parameters of four blood pressure long-term level patterns

Class number	AIC	BIC	Long-term levels class (%)							Posterior probability						
			class1	class2	class3	class4	class5	class6	class7	class1	class2	class3	class4	class5	class6	class7
**SBP**																
1	541484.85	541514.01	100							1.00						
2	531650.31	531708.62	68.05	31.95						0.91	0.87					
**3**	**528219.25**	**528306.71**	**18.67**	**69.99**	**11.34**					**0.83**	**0.87**	**0.87**				
4	527085.08	527201.70	7.49	62.11	26.96	3.44				0.82	0.83	0.80	0.87			
5	526491.52	526637.29	7.68	62.32	23.68	2.66	3.65			0.83	0.82	0.75	0.86	0.74		
6	525930.11	526105.04	3.16	30.6	52.35	8.27	4.31	1.30		0.84	0.76	0.75	0.75	0.75	0.88	
7	525675.45	525879.53	3.21	31.24	51.6	1.16	7.91	3.74	1.14	0.84	0.76	0.75	0.76	0.74	0.74	0.89
**DBP**																
1	492287.89	492317.05	100							1.00						
2	480311.29	480369.60	57.91	42.09						0.91	0.88					
**3**	**475927.94**	**476015.41**	**25.42**	**64.38**	**10.21**					**0.86**	**0.88**	**0.88**				
4	474533.58	474650.20	10.7	33.75	51.34	4.22				0.84	0.81	0.81	0.88			
5	473750.93	473896.70	8.27	7.33	41.95	42.11	0.33			0.83	0.85	0.79	0.79	0.94		
6	473356.22	473531.16	40.07	7.84	43.74	5.82	2.21	0.31		0.79	0.83	0.79	0.79	0.75	0.95	
7	473070.97	473275.06	3.55	24.11	50.33	17.00	2.81	1.94	0.25	0.83	0.76	0.74	0.71	0.80	0.77	0.94
**mid-BP (calculated as [SBP+DBP]/2)**																
1	502355.84	502384.99	100							1.00						
2	491129.64	491187.95	69.73	30.27						0.92	0.87					
**3**	**487239.69**	**487327.15**	**31.08**	**60.85**	**8.07**					**0.84**	**0.86**	**0.89**				
4	485835.30	485951.93	9.46	61.93	25.61	3.00				0.83	0.84	0.82	0.89			

5	485265.91	485411.68	10.71	5.27	64.52	17.34	2.17			0.83	0.73	0 83	0.74	0.88		
6	484571.85	484746.78	5.67	46.0	37.78	5.56	4.04	0.96		0.83	0.78	0.74	0.78	0.75	0.89	
7	484345.19	484549.27	5.81	46.64	4.52	37.10	1.74	3.22	0.91	0.83	0.78	0.74	0.73	0.71	0.71	0.88
**mean arterial BP (calculated as [**(1/3)×SBP+(2/3)×DBP**])**																
1	494910.34	494939.50	100							1.00						
2	483290.56	483348.87	70.16	29.84						0.93	0.87					
**3**	**478873.97**	**478961.43**	**33.44**	**59.30**	**7.26**					**0.85**	**0.87**	**0.89**				
4	477237.83	477354.45	21.75	63.91	13.45	0.90				0.83	0.85	0.85	0.92			
5	476660.16	476805.93	21.37	6.56	7.08	64.23	0.76			0.83	0.73	0.78	0.84	0.91		
6	475731.52	475906.45	7.46	48.77	36.06	4.79	2.74	0.18		0.83	0.79	0.77	0.83	0.77	0.95	
7	475368.55	475572.64	6.83	45.10	38.18	5.50	0.80	3.52	0.06	0.83	0.78	0.75	0.79	0.89	0.75	1.00

AIC – Akaike information criterion, BIC – Bayesian information criterion, DBP – diastolic blood pressure, SBP – systolic blood pressure

Table S3. Values of each distinct long-term levels of blood pressure in four measurements

Values		Long-term levels*		
**SBP, mm Hg**				
		**Normal SBP**	**High-normal SBP**	**Uncontrolled SBP**
Overall	Number of measurements	14143	48405	9547
	SBP (mean±SD)	120±10	132±9	146±14
	SBP (median [IQR])	120 [114, 126]	132 [126, 137]	143 [136, 153]
0 to 3 months	Number of measurements	3631	11874	2320
	SBP (mean±SD)	120±11	132±10	147±14
	SBP (median [IQR])	120 [114, 126]	132 [126, 137]	145 [137, 155]
3 to 6 months	Number of measurements	3486	12084	2378
	SBP (mean±SD)	120±10	132±9	146±14
	SBP (median [IQR])	120 [113, 126]	132 [126, 136]	143 [136, 153]
6 to 9 months	Number of measurements	34742	12004	2419
	SBP (mean±SD)	120±10	132±9	145±13
	SBP (median [IQR])	120[115, 126]	132 [127, 136]	143 [136, 152]
9 to 12 months	Number of measurements	3552	12443	2430
	SBP (mean±SD)	121±10	132±9	145±14
	SBP (median [IQR])	121 [115, 127]	132 [126, 137]	142 [136, 152]
**DBP, mm Hg**				
		**Low-normal DBP**	**Normal DBP**	**Uncontrolled DBP**
Overall	Number of measurements	18984	44756	8355
	DBP (mean±SD)	72±7	81±7	90±9
	DBP (median [IQR])	72 [68, 76]	80 [76, 85]	89 [85, 95]
0 to 3 months	Number of measurements	4676	11084	2065
	DBP (mean±SD)	72±7	81±7	92±10
	DBP (median [IQR])	72 [68, 76]	80 [77, 85]	90 [86, 98]
3 to 6 months	Number of measurements	4792	11027	2129
	DBP (mean±SD)	72±7	81±7	90±9
	DBP (median [IQR])	72 [68,76]	80 [77,85]	89 [85, 95]
6 to 9 months	Number of measurements	4613	11173	2111
	DBP (mean±SD)	72±7	80±7	90±9
	DBP (median [IQR])	72 [68, 76]	80[76, 85]	89 [85, 95]
9 to 12 months	Number of measurements	4903	11472	2050
	DBP (mean±SD)	72±7	81±7	89±9
	DBP (median [IQR])	72 [68, 76]	80 [76, 85]	88 [84, 94]
**mid-BP, mm Hg (calculated as [SBP+DBP]/2)**				
		**Low-stable mid-BP**	**Middle-stable mid-BP**	**High-stable mid-BP**
Overall	Number of measurements	22636	42637	6822

	mid-BP (mean±SD)	99±7	107±7	118±10
	mid-BP (median [IQR])	100 [95, 103]	107 [103, 111]	116 [110, 124]
0 to 3 months	Number of measurements	5692	10432	1701
	mid-BP (mean±SD)	99±7	107±8	120±11
	mid-BP (median [IQR])	100 [95, 103]	107[102, 112]	119 [112, 127]
3-6 months	Number of measurements	5609	10617	1722
	mid-BP (mean±SD)	98±7	107±7	118±10
	mid-BP (median [IQR])	99 [94, 102]	106 [103, 110]	116 [111, 124]
6 to 9 months	Number of measurements	5546	10611	1740
	mid-BP (mean±SD)	99±7	107±7	117±10
	mid-BP (median [IQR])	100 [95, 103]	106 [103, 110]	116 [110,123]
9 to 12 months	Number of measurements	5789	10977	1659
	mid-BP (mean±SD)	99±7	107±7	117±10
	mid-BP (median [IQR])	100 [95, 103]	107 [103, 111]	115 [110, 123]
**mean arterial BP, mm Hg (calculated as [(1/3)×SBP+(2/3)×DBP])**				
		**Low-stable mean arterial BP**	**Middle-stable mean arterial BP**	**High-stable mean arterial BP**
Overall	Number of measurements	6145	24439	41511
	mean arterial BP (mean±SD)	91±7	99±7	109±10
	mean arterial BP (median [IQR])	91 [87, 95]	98 [95, 102]	108 [102, 115]
0 to 3 months	Number of measurements	6103	10160	1562
	mean arterial BP (mean±SD)	91±7	99±7	112±11
	mean arterial BP (median [IQR])	91 [87, 95]	99 [94,103]	110 [104, 118]
3 to 6 months	Number of measurements	6128	10266	1554
	mean arterial BP (mean±SD)	90±7	99±7	109±10
	mean arterial BP (median [IQR])	91 [87, 94]	98 [95, 102]	107 [102, 115]
6 to 9 months	Number of measurements	5966	10388	1543
	mean arterial BP (mean±SD)	90±6	99±7	109±10
	mean arterial BP (median [IQR])	91 [87, 94]	98 [94, 102]	107 [102, 114]
9 to 12 months	Number of measurements	6242	10697	1486
	mean arterial BP (mean±SD)	91±7	99±7	108±10
	mean arterial BP (median [IQR])	91 [87, 95]	98 [95, 102]	107 [102, 113]

DBP – diastolic blood pressure, IQR – interquartile range, SBP – systolic blood pressure, SD – standard deviation.

* Long-term BP groups were constructed based on the latent clusters identified using the group-based trajectory model.

Table S4. Risk of clinical outcomes in long-term normal vs. high-normal SBP levels within a 36-month follow-up period

	**Normal SBP (N=2120)***	**High-normal SBP (N=7949)***
**Recurrent stroke**		
Crude model: HR (95% CI)†	0.93 (0.79–1.08)	Ref.
Adjusted model 1: HR (95% CI)†	0.94 (0.80–1.10)	Ref.
Adjusted model 2: HR (95% CI)†	0.94 (0.80–1.10)	Ref.
Weighted model: HR (95% CI)†	0.92 (0.79–1.08)	Ref.
**MACE**		
Crude model: HR (95% CI)†	0.84 (0.75–0.94) ‡	Ref.
Adjusted model 1: HR (95% CI)†	0.85 (0.76–0.95) ‡	Ref.
Adjusted model 2: HR (95% CI)†	0.86 (0.77–0.96) ‡	Ref.
Weighted model: HR (95% CI)†	0.86 (0.77–0.96) ‡	Ref.

95% CI – 95% confidence interval, HR – hazard ratio, MACE – major adverse cardiovascular events, SBP – systolic blood pressure.

* Long-term SBP groups were constructed based on the latent clusters identified using the group-based trajectory model.

† Crude model: without any adjustment. Adjusted Model 1: adjusted for age and sex. Adjusted model 2: adjusted for sex, age, ethnic groups, marital status, education levels, jobs, insurance payment, smoking, alcohol drinking, hypertension, diabetes, hyperlipidemia, atrial fibrillation, carotid artery stenosis, chronic kidney disease, hemiplegia, transient ischemic attack, previous stroke, antihypertensive drugs, antidiabetic drugs, statins, endovascular therapy, thrombolysis, anticoagulant therapy, and antiplatelet therapy. Weighted model: weighted by propensity score overlap weights.

‡ *P<0.05* was considered statistically significant.

Table S5. Restricted mean survival time of different long-term SBP levels by age for recurrent stroke and MACE

**Outcome**	**Long-term levels***	**Age group**							
		**45**	**50**	**55**	**60**	**65**	**70**	**75**	**80**
**Normal SBP vs. Uncontrolled SBP**									
Recurrent Stroke	Normal SBP	20.12 (18.03–22.21)	16.25 (14.66–17.83)	12.09 (10.89–13.29)	8.60 (7.67–9.53)	5.72 (5.01–6.42)	3.55 (3.03–4.06)	1.99 (1.64–2.33)	0.95 (0.72–1.17)
Recurrent Stroke	High-normal SBP	13.00 (10.52–15.47)	9.75 (7.98–11.53)	6.75 (5.66–7.85)	4.58 (3.78–5.37)	3.09 (2.72–3.45)	1.92 (1.63–2.21)	1.09 (0.88–1.31)	0.57 (0.45–0.69)
**Event-free survival time†**		**7.12 (3.88–10.36)**	**6.49 (4.11–8.88)**	**5.34 (3.71–6.97)**	**4.03 (2.80–5.25)**	**2.63 (1.84–3.43)**	**1.62 (1.03–2.22)**	**0.89 (0.49–1.30)**	**0.38 (0.12–0.63)**
MACE	Normal SBP	12.83 (11.35–14.31)	9.11 (8.12–10.11)	5.55 (4.92–6.18)	3.11 (2.76–3.46)	1.58 (1.40–1.77)	0.68 (0.57–0.79)	0.23 (0.19–0.27)	0.06 (0.05–0.08)
MACE	High-normal SBP	7.03 (5.56–8.50)	4.45 (3.63–5.27)	2.27 (1.83–2.70)	1.14 (0.89–1.39)	0.58 (0.51–0.66)	0.27 (0.23–0.31)	0.11 (0.08–0.13)	0.04 (0.03–0.05)
**Event-free survival time†**		**5.80 (3.71–7.89)**	**4.66 (3.37–5.95)**	**3.28 (2.52–4.05)**	**1.97 (1.54–2.40)**	**1.00 (0.80–1.20)**	**0.41 (0.30–0.53)**	**0.13 (0.08–0.17)**	**0.02 (0.00–0.04)**
**High-normal SBP vs. Uncontrolled SBP**									
Recurrent Stroke	Normal SBP	18.52 (17.40–19.64)	14.70 (13.85–15.55)	10.80 (10.18–11.42)	7.71 (7.26–8.16)	5.27 (4.97–5.57)	3.43 (3.21–3.66)	2.05 (1.90–2.21)	1.13 (1.03–1.23)
Recurrent Stroke	High-normal SBP	13.00 (10.52–15.47)	9.75 (7.98–11.53)	6.75 (5.66–7.85)	4.58 (3.78–5.37)	3.09 (2.72–3.45)	1.92 (1.63–2.21)	1.09 (0.88–1.31)	0.57 (0.45–0.69)
**Event-free survival time†**		**5.52 (2.81–8.24)**	**4.95 (2.98–6.92)**	**4.05 (2.79–5.31)**	**3.13 (2.22–4.04)**	**2.19 (1.71–2.66)**	**1.51 (1.14–1.87)**	**0.96 (0.69–1.23)**	**0.56 (0.40–0.72)**

MACE	Normal SBP	11.26 (10.50–12.02)	7.73 (7.26–8.19)	4.56 (4.27–4.84)	2.45 (2.28–2.62)	1.21 (1.14–1.27)	0.54 (0.50–0.58)	0.20 (0.18–0.21)	0.06 (0.05–0.07)
MACE	High-normal SBP	7.03 (5.56–8.50)	4.45 (3.63–5.27)	2.27 (1.83–2.70)	1.14 (0.89–1.39)	0.58 (0.51–0.66)	0.27 (0.23–0.31)	0.11 (0.08–0.13)	0.04 (0.03–0.05)
**Event-free survival time†**		**4.23 (2.57–5.89)**	**3.28 (2.34–4.22)**	**2.29 (1.77–2.81)**	**1.31 (1.01–1.61)**	**0.62 (0.52–0.72)**	**0.27 (0.21–0.33)**	**0.09 (0.06–0.12)**	**0.02 (0.01–0.03)**
**Normal SBP vs. High-normal SBP**									
Recurrent Stroke	Normal SBP	20.12 (18.03–22.21)	16.25 (14.66–17.83)	12.09 (10.89–13.29)	8.60 (7.67–9.53)	5.72 (5.01–6.42)	3.55 (3.03–4.06)	1.99 (1.64–2.33)	0.95 (0.72–1.17)
Recurrent Stroke	High-normal SBP	18.52 (17.40–19.64)	14.70 (13.85–15.55)	10.80 (10.18–11.42)	7.71 (7.26–8.16)	5.27 (4.97–5.57)	3.43 (3.21–3.66)	2.05 (1.90–2.21)	1.13 (1.03–1.23)
**Event-free survival time†**		**1.60 (–0.77, 3.97)**	**1.55 (–0.25, 3.35)**	**1.29 (–0.06, 2.64)**	**0.89 (–0.14, 1.93)**	**0.45 (–0.32, 1.21)**	**0.12 (–0.45, 0.68)**	**–0.07 (–0.44, 0.31)**	**–0.18 (–0.43, 0.06)**
MACE	Normal SBP	11.26 (10.50–12.02)	7.73 (7.26–8.19)	4.56 (4.27–4.84)	2.45 (2.28–2.62)	1.21 (1.14–1.27)	0.54 (0.50–0.58)	0.20 (0.18–0.21)	0.06 (0.05–0.07)
MACE	High-normal SBP	12.83 (11.35–14.31)	9.11 (8.12–10.11)	5.55 (4.92–6.18)	3.11 (2.76–3.46)	1.58 (1.40–1.77)	0.68 (0.57–0.79)	0.23 (0.19–0.27)	0.06 (0.05–0.08)
**Event-free survival time†**		**1.57 (–0.10, 3.23)**	**1.38 (0.29–2.48)**	**0.99 (0.30–1.68)**	**0.66 (0.27–1.05)**	**0.38 (0.18–0.58)**	**0.14 (0.03–0.26)**	**0.03 (–0.10, 0.08)**	**0.00 (–0.01, 0.02)**

MACE – major cardiovascular events, SBP – systolic blood pressure.

* Long-term SBP groups were constructed based on the latent clusters identified using the group-based trajectory model.

† Unit of event-free survival time: years.

Table S6. Sensitivity analysis of association between long-term SBP levels and the risk of recurrent stroke and MACE

Groups*	Recurrent stroke		MACE	
	HR/SHR (95% CI)	*P*	HR/SHR (95% CI)	*P*
**Sensitivity analysis 1: Regarding non-stroke-related or non-MACE-related death as the competitive events†**				
Uncontrolled SBP	1.00 (Ref.)		1.00 (Ref.)	
Normal SBP	0.76 (0.61–0.95)	0.014‡	0.84 (0.72–0.97)	0.021‡
High-normal SBP	0.82 (0.69–0.99)	0.034‡	0.97 (0.85–1.10)	0.600
**Sensitivity analysis 2: Including hospital level (tertiary, secondary, or community primary) as a random effect in the Cox models†**				
Uncontrolled SBP	1.00 (Ref.)		1.00 (Ref.)	
Normal SBP	0.73 (0.58–0.92)	0.007‡	0.83 (0.70–0.98)	0.027‡
High-normal SBP	0.78 (0.59–1.03)	0.077	1.03 (0.84–1.26)	0.780
**Sensitivity analysis 3: Excluding participants whose posterior probability of assignment to their most likely group was <0.70†**				
Uncontrolled SBP	1.00 (Ref.)		1.00 (Ref.)	
Normal SBP	0.61 (0.46–0.80)	<0.001‡	0.74 (0.61–0.90)	0.002‡
High-normal SBP	0.78 (0.64–0.95)	0.015‡	0.92 (0.80–1.07)	0.274
**Sensitivity analysis 4: Excluding patients with prevalent hemiplegia†**				
Uncontrolled SBP	1.00 (Ref.)		1.00 (Ref.)	
Normal SBP	0.68 (0.54–0.86)	0.001‡	0.76 (0.64–0.90)	0.002‡
High-normal SBP	0.77 (0.65–0.93)	0.005‡	0.89 (0.78–1.02)	0.095
**Sensitivity analysis 5: Excluding patients with previous stroke or transient ischemic attack†**				
Uncontrolled SBP	1.00 (Ref.)		1.00 (Ref.)	
Normal SBP	0.68 (0.54–0.86)	0.001‡	0.76 (0.64–0.90)	0.001‡
High-normal SBP	0.78 (0.65–0.93)	0.006‡	0.89 (0.78–1.02)	0.091
**Sensitivity analysis 6: Excluding participants with < 4 measurement of SBP†**				
Uncontrolled SBP	1.00 (Ref.)		1.00 (Ref.)	
Normal SBP	0.68 (0.54–0.86)	0.001‡	0.79 (0.67–0.95)	0.009‡
High-normal SBP	0.76 (0.64–0.92)	0.004‡	0.91 (0.80–1.05)	0.189
**Sensitivity analysis 7: Excluding participants with measurement of SBP failing to cover the four measurement windows†**				
Uncontrolled SBP	1.00 (Ref.)		1.00 (Ref.)	
Normal SBP	0.68 (0.51–0.91)	0.010‡	0.73 (0.58–0.92)	0.008‡
High-normal SBP	0.73 (0.58–0.92)	0.008‡	0.85 (0.68–1.06)	0.140
**Sensitivity analysis 8: Using the inverse propensity of treatment weighting method to balance baseline covariates†**				
Uncontrolled SBP	1.00 (Ref.)		1.00 (Ref.)	
Normal SBP	0.68 (0.54–0.86)	0.001‡	0.78 (0.66–0.93)	0.005‡

High-normal SBP	0.77 (0.64–0.92)	0.005‡	0.94 (0.82–1.08)	0.391
**Sensitivity analysis 9: Further adjusting the mean SBP within exposure window and four measurement windows, respectively†**				
**Adjusting the mean SBP within the exposure window**				
Uncontrolled SBP	1.00 (Ref.)		1.00 (Ref.)	
Normal SBP	0.52 (0.30–0.89)	0.017‡	0.74 (0.50–1.08)	0.121
High-normal SBP	0.64 (0.47–0.89)	0.007‡	0.84 (0.67–1.07)	0.154
**Adjusting the mean SBP within the measurement windows of 0 to 3 months**				
Uncontrolled SBP	1.00 (Ref.)		1.00 (Ref.)	
Normal SBP	0.58 (0.40–0.85)	0.005‡	0.74 (0.58–0.95)	0.017‡
High-normal SBP	0.67 (0.52–0.86)	0.002‡	0.85 (0.72–1.01)	0.073
**Adjusting the mean SBP within the measurement windows of 3 to 6 months**				
Uncontrolled SBP	1.00 (Ref.)		1.00 (Ref.)	
Normal SBP	0.56 (0.41–0.78)	0.001‡	0.67 (0.52–0.85)	0.001‡
High-normal SBP	0.68 (0.54–0.85)	0.001‡	0.81 (0.69–0.96)	0.013‡
**Adjusting the mean SBP within the measurement windows of 6 to 9 months**				
Uncontrolled SBP	1.00 (Ref.)		1.00 (Ref.)	
Normal SBP	0.73 (0.51–1.03)	0.075	0.78 (0.61–0.99)	0.039‡
High-normal SBP	0.75 (0.60–0.94)	0.013‡	0.85 (0.72–1.00)	0.052
**Adjusting the mean SBP within the measurement windows of 9 to 12 months**				
Uncontrolled SBP	1.00 (Ref.)		1.00 (Ref.)	
Normal SBP	0.60 (0.42–0.85)	0.004‡	0.79 (0.62–1.02)	0.067
High-normal SBP	0.68 (0.54–0.85)	0.001‡	0.89 (0.76–1.06)	0.186

95% CI – 95% confidence interval, HR – hazard ratio, MACE – major adverse cardiovascular events, SBP – systolic blood pressure, SHR – subdistribution hazard ratio, the effect size for competing risk analysis in Sensitivity analysis.

* Long-term SBP groups were constructed based on the latent clusters identified using the group-based trajectory model.

† Sensitivity analysis 1 to 7 were weighting by overlap propensity score, respectively. In sensitivity analysis 9, these models were full adjusted models, which were adjusted for sex, age, ethnic group, marital status, education levels, jobs, insurance payment, smoking, alcohol drinking, hypertension, diabetes, hyperlipidemia, atrial fibrillation, carotid artery stenosis, chronic kidney disease, hemiplegia, transient ischemic attack, previous stroke, statins, antihypertensive drugs, antidiabetic drugs, endovascular therapy, thrombolysis, anticoagulant therapy, and antiplatelet therapy.

‡ $P<0.05$ was considered statistically significant.

Table S7. Association between long-term SBP levels and ischemic heart disease, heart failure, and hemorrhagic transformation during a 36-month follow-up period

	**Normal SBP (N=2120)***	**High-normal SBP (N=7949)***	**Uncontrolled SBP (N=1288)***
**Ischemic heart disease**			
Number of events	174	750	103
Incidence rate (95% CI)†	52.7 (45.2–61.2)	58.2 (54.1–62.5)	51.1 (41.7–61.9)
Crude model: HR (95% CI)‡	1.02 (0.80–1.31)	1.15 (0.93–1.41)	Ref.
Adjusted model 1: HR (95% CI)‡	1.06 (0.83–1.24)	1.10 (0.90–1.35)	Ref.
Adjusted model 2: HR (95% CI)‡	0.89 (0.70–1.15)	1.03 (0.84–1.27)	Ref.
Weighted model: HR (95% CI)‡	0.93 (0.71–1.21)	1.04 (0.84–1.30)	Ref.
**Heart failure**			
Number of events	64	359	58
Incidence rate (95% CI)†	19.4 (14.9–24.8)	27.9 (25.0–30.9)	28.8 (21.8–37.2)
Crude model: HR (95% CI)‡	0.67 (0.47–0.95)§	0.98 (0.74–1.29)	Ref.
Adjusted model 1: HR (95% CI)‡	0.71 (0.49–1.01)	0.94 (0.71–1.24)	Ref.
Adjusted model 2: HR (95% CI)‡	0.63 (0.44–0.91)§	0.92 (0.69–1.22)	Ref.
Weighted model: HR (95% CI)‡	0.68 (0.46–1.01)	0.98 (0.73–1.31)	Ref.
**Hemorrhagic transformation**			
Number of events	12	50	19
Incidence rate (95% CI)†	3.4 (1.7–5.9)	3.5 (2.6–4.6)	8.7 (5.2–13.5)
Crude model: HR (95% CI)‡	0.39 (0.19–0.80)§	0.41 (0.24–0.70)§	Ref.
Adjusted model 1: HR (95% CI)‡	0.43 (0.21–0.89)§	0.43 (0.25–0.73)§	Ref.
Adjusted model 2: HR (95% CI)‡	0.36 (0.17–0.77)§	0.39 (0.23–0.67)§	Ref.
Weighted model: HR (95% CI)‡	0.24 (0.10–0.55)§	0.44 (0.25–0.79)§	Ref.

95% CI – 95% confidence interval, HR – hazard ratio, MACE – major adverse cardiovascular events, SBP – systolic blood pressure.

* Long-term SBP groups were constructed based on the latent clusters identified using the group-based trajectory model.

† Unit of incidence rate: /1000 person-years.

‡ Crude model: without any adjustment. Adjusted Model 1: adjusted for age and sex. Adjusted model 2: adjusted for sex, age, ethnic groups, marital status, education levels, jobs, insurance payment, smoking, alcohol drinking, hypertension, diabetes, hyperlipidemia, atrial fibrillation, carotid artery stenosis, chronic kidney disease, hemiplegia, transient ischemic attack, previous stroke, antihypertensive drugs, antidiabetic drugs, statins, endovascular therapy, thrombolysis, anticoagulant therapy, and antiplatelet therapy. Weighted model: weighted by propensity score overlap weights.

§ $P<0.05$ was considered statistically significant.

Table S8. Association between long-term DBP levels and clinical outcomes during a 36-month follow-up period

	Low-normal DBP (N=2887)*	Normal DBP (N=7311)*	Uncontrolled DBP (N=1159)*
**Recurrent stroke**			
Number of events	343	771	110
Incidence rate (95% CI)†	43.3 (38.8–48.1)	38.2 (35.5–41.0)	34.3 (28.2–41.4)
Crude model: HR (95% CI)‡	1.29 (1.06–1.58)	1.10 (0.91–1.33)	Ref.
Adjusted model 1: HR (95% CI)‡	0.84 (0.68–1.04)	0.87 (0.72–1.06)	Ref.
Adjusted model 2: HR (95% CI)‡	0.77 (0.62–0.96)	0.82 (0.68–1.00)§	Ref.
Weighted model: HR (95% CI)‡	0.86 (0.67–1.11)	0.88 (0.71–1.10)	Ref.
**MACE**			
Number of events	749	1479	193
Incidence rate (95% CI)†	101.7 (94.6–109.3)	77.4 (73.5–81.5)	62.3 (53.8–71.7)
Crude model: HR (95% CI)‡	1.58 (1.36–1.84)	1.20 (1.04–1.38)§	Ref.
Adjusted model 1: HR (95% CI)‡	0.95 (0.81–1.11)	0.91 (0.78–1.05)	Ref.
Adjusted model 2: HR (95% CI)‡	0.78 (0.67–0.92)§	0.84 (0.72–0.97)§	Ref.
Weighted model: HR (95% CI)‡	0.90 (0.74–1.08)	0.90 (0.77–1.06)	Ref.

95% CI – 95% confidence interval, DBP –diastolic blood pressure, HR – hazard ratio, MACE – major adverse cardiovascular events.

* Long-term DBP groups were constructed based on the latent clusters identified using the group-based trajectory model.

† Unit of incidence rate: /1000 person-years.

‡ Crude model: without any adjustment. Adjusted Model 1: adjusted for age and sex. Adjusted model 2: adjusted for sex, age, ethnic groups, marital status, education levels, jobs, insurance payment, smoking, alcohol drinking, hypertension, diabetes, hyperlipidemia, atrial fibrillation, carotid artery stenosis, chronic kidney disease, hemiplegia, transient ischemic attack, previous stroke, antihypertensive drugs, antidiabetic drugs, statins, endovascular therapy, thrombolysis, anticoagulant therapy, and antiplatelet therapy. Weighted model: weighted by propensity score overlap weights.

§ $P<0.05$ was considered statistically significant.

Table S9. Association between long-term mid-BP levels and clinical outcomes during a 36-month follow-up period

	**Low-stable mid-BP (N=3530)***	**Middle-stable mid-BP (N=6911)***	**High-stable mid-BP (N=916)***
**Recurrent stroke**			
Number of events	397	727	100
Incidence rate (95% CI)†	41.1 (37.1–45.3)	38.0 (35.3–40.9)	39.8 (32.3–48.3)
Crude model: HR (95% CI)‡	1.09 (0.88–1.34)	1.01 (0.83–1.24)	Ref.
Adjusted model 1: HR (95% CI)‡	0.88 (0.71–1.09)	0.88 (0.72–1.08)	Ref.
Adjusted model 2: HR (95% CI)‡	0.81 (0.65–1.01)	0.84 (0.69–1.04)	Ref.
Weighted model: HR (95% CI)‡	0.84 (0.67–1.06)	0.90 (0.73–1.11)	Ref.
**MACE**			
Number of events	801	1450	170
Incidence rate (95% CI)†	87.9 (81.9–94.2)	80.4 (76.3–84.6)	70.4 (60.2–81.8)
Crude model: HR (95% CI)‡	1.25 (1.06–1.46)	1.15 (0.98–1.33)	Ref.
Adjusted model 1: HR (95% CI)‡	0.97 (0.83–1.14)	0.98 (0.84–1.14)	Ref.
Adjusted model 2: HR (95% CI)‡	0.82 (0.70–0.97)§	0.92 (0.79–1.08)	Ref.
Weighted model: HR (95% CI)‡	0.88 (0.74–1.05)	0.97 (0.83–1.14)	Ref.

95% CI – 95% confidence interval, HR – hazard ratio, MACE – major adverse cardiovascular events, mid-BP – mid-blood pressure.

* Long-term mid-blood groups were constructed based on the latent clusters identified using the group-based trajectory model.

† Unit of incidence rate: /1000 person-years.

‡ Crude model: without any adjustment. Adjusted Model 1: adjusted for age and sex. Adjusted model 2: adjusted for sex, age, ethnic groups, marital status, education levels, jobs, insurance payment, smoking, alcohol drinking, hypertension, diabetes, hyperlipidemia, atrial fibrillation, carotid artery stenosis, chronic kidney disease, hemiplegia, transient ischemic attack, previous stroke, antihypertensive drugs, antidiabetic drugs, statins, endovascular therapy, thrombolysis, anticoagulant therapy, and antiplatelet therapy. Weighted model: weighted by propensity score overlap weights.

§ $P<0.05$ was considered statistically significant.

Table S10. Association between long-term mean arterial BP levels and clinical outcomes during a 36-month follow-up period

	**Low-stable mean arterial BP (N=3798)***	**Middle-stable mean arterial BP (N=6735)***	**High-stable mean arterial BP (N=824)***
**Recurrent stroke**			
Number of events	427	709	88
Incidence rate (95% CI)†	40.8 (37.0–44.8)	38.1 (35.4–41.0)	39.2 (31.5–48.3)
Crude model: HR (95% CI)‡	1.09 (0.87–1.35)	1.01 (0.81–1.25)	Ref.
Adjusted model 1: HR (95% CI)‡	0.81 (0.65–1.02)	0.85 (0.69–1.05)	Ref.
Adjusted model 2: HR (95% CI)‡	0.76 (0.60–0.95)§	0.82 (0.66–1.01)	Ref.
Weighted model: HR (95% CI)‡	0.81 (0.63–1.03)	0.89 (0.71–1.11)	Ref.
**MACE**			
Number of events	897	1381	143
Incidence rate (95% CI)†	91.4 (85.5–97.6)	78.6 (74.5–82.8)	65.8 (55.5–77.5)
Crude model: HR (95% CI)‡	1.38 (1.17–1.64)§	1.20 (1.02–1.42)§	Ref.
Adjusted model 1: HR (95% CI)‡	0.98 (0.82–1.16)	0.99 (0.84–1.17)	Ref.
Adjusted model 2: HR (95% CI)‡	0.84 (0.71–1.00)§	0.94 (0.80–1.11)	Ref.
Weighted model: HR (95% CI)‡	0.90 (0.74–1.08)	0.99 (0.83–1.18)	Ref.

95% CI – 95% confidence interval, HR – hazard ratio, MACE – major adverse cardiovascular events, mean arterial BP – mean arterial blood pressure.

* Long-term mean arterial BP groups were constructed based on the latent clusters identified using the group-based trajectory model.

† Unit of incidence rate: /1000 person-years.

‡ Crude model: without any adjustment. Adjusted Model 1: adjusted for age and sex. Adjusted model 2: adjusted for sex, age, ethnic groups, marital status, education levels, jobs, insurance payment, smoking, alcohol drinking, hypertension, diabetes, hyperlipidemia, atrial fibrillation, carotid artery stenosis, chronic kidney disease, hemiplegia, transient ischemic attack, previous stroke, antihypertensive drugs, antidiabetic drugs, statins, endovascular therapy, thrombolysis, anticoagulant therapy, and antiplatelet therapy. Weighted model: weighted by propensity score overlap weights.

§ $P<0.05$ was considered statistically significant.
